# Atopic eczema and major cardiovascular outcomes: A systematic review and meta-analysis of population-based studies

**DOI:** 10.1016/j.jaci.2018.11.030

**Published:** 2019-05

**Authors:** Anna Ascott, Amy Mulick, Ashley M. Yu, David Prieto-Merino, Morten Schmidt, Katrina Abuabara, Liam Smeeth, Amanda Roberts, Sinéad M. Langan

**Affiliations:** a Royal Sussex County Hospital, Eastern Road, Brighton, United Kingdom; b Department of Non-Communicable Disease Epidemiology, London School of Hygiene and Tropical Medicine, London, United Kingdom; c Faculty of Medicine, University of Ottawa, Ottawa, Ontario, Canada; d Department of Clinical Epidemiology, Aarhus University Hospital, Denmark Department of Cardiology, Regional Hospital West Jutland, Herning, Denmark; e Program for Clinical Research, Department of Dermatology, University of California, San Francisco School of Medicine, San Francisco, Calif; f Nottingham Support Group for Carers of Children with Eczema, Nottingham, United Kingdom

**Keywords:** Atopic eczema, atopic dermatitis, angina, myocardial infarction, heart failure, stroke, ischemic stroke, cardiovascular death, cardiovascular outcomes, risk factors, CrI, Credibility interval, CVD, Cardiovascular disease, GRADE, Grading of Recommendations Assessment, Development and Evaluation, HR, Hazard ratio, IRR, Incidence rate ratio, OR, Odds ratio, PI, Prediction interval, PRISMA, Preferred Reporting Items for Systematic Review and Meta-Analysis, PROSPERO, Prospective Register of Systematic Reviews, RR, Relative risk

## Abstract

**Background:**

Atopic eczema is a common inflammatory skin disease. Various inflammatory conditions have been linked to cardiovascular disease, a major cause of global mortality and morbidity.

**Objective:**

We sought to systematically review and meta-analyze population-based studies assessing associations between atopic eczema and specific cardiovascular outcomes.

**Methods:**

MEDLINE, Embase, and Global Health were searched from inception to December 2017. We obtained pooled estimates using random-effects meta-analyses. We used a multivariate Bayesian meta-regression model to estimate the slope of effect of increasing atopic eczema severity on cardiovascular outcomes.

**Results:**

Nineteen relevant studies were included. The effects of atopic eczema reported in cross-sectional studies were heterogeneous, with no evidence for pooled associations with angina, myocardial infarction, heart failure, or stroke. In cohort studies atopic eczema was associated with increased risk of myocardial infarction (n = 4; relative risk [RR], 1.12; 95% CI, 1.00-1.25), stroke (n = 4; RR, 1.10; 95% CI, 1.03-1.17), ischemic stroke n = 4; RR, 1.17; 95% CI, 1.14-1.20), angina (n = 2; RR, 1.18; 95% CI, 1.13-1.24), and heart failure (n = 2; RR, 1.26; 95% CI, 1.05-1.51). Prediction intervals were wide for myocardial infarction and stroke.

The risk of cardiovascular outcomes appeared to increase with increasing severity (mean RR increase between severity categories, 1.15; 95% credibility interval, 1.09-1.21; uncertainty interval, 1.04-1.28).

**Conclusion:**

Significant associations with cardiovascular outcomes were more common in cohort studies but with considerable between-study heterogeneity. Increasing atopic eczema severity was associated with increased risk of cardiovascular outcomes. Improved awareness among stakeholders regarding this small but significant association is warranted.

Atopic eczema (also known as atopic dermatitis^1^) is an inflammatory skin disease traditionally considered a disease of childhood. However, atopic eczema can affect around 10% of adults,^2^, ^3^ and its global prevalence has increased.^4^ Concurrently, cardiovascular disease (CVD) is a major cause of mortality and morbidity globally and has been linked to various chronic inflammatory conditions.^5^

Increasing evidence supports an association between atopic eczema and CVD. Mechanistic studies suggest that platelet dysfunction and decreased fibrinolysis can contribute to increased clotting in patients with atopic eczema.^6^, ^7^ The impaired skin barrier is known to be more susceptible to acute and chronic local infection, contributing to inflammation. In addition, treatments used for atopic eczema can increase cardiovascular risk.^8^, ^9^

Epidemiologic studies have inconsistently linked atopic eczema to cardiovascular risk factors and outcomes across different populations.^10^, ^11^, ^12^, ^13^ Previous systematic reviews have found an association between atopic eczema and risk factors for CVD, including increased body mass index^14^ and childhood type 1 diabetes^15^; however, another review concluded that there was no association between atopic eczema and most cardiovascular outcomes.^16^ Of note, this review did not consider atopic eczema severity.

We conducted a systematic review and meta-analysis of the associations between adult atopic eczema and adverse cardiovascular outcomes overall and according to atopic eczema severity.

## Methods

The protocol of this systematic review was registered before the study's start with the International Prospective Register of Systematic Reviews (PROSPERO; CRD42017060359) and published in full.^17^ The study is reported in line with Preferred Reporting Items for Systematic Review and Meta-Analysis (PRISMA).^18^

### Data sources and searches

Comprehensive search strategies were cocreated by a medical professional and librarian and reviewed by all authors. We used Ovid to search MEDLINE, Embase, and Global Health from their dates of inception to December 16, 2017. Embase was searched from 1947, Global Health from 1910, and Ovid MEDLINE from 1946. Exclusion filters and limits were not applied to minimize the risk that eligible studies could be inadvertently excluded. The Ovid MEDLINE search strategy is available to view (see the supplementary material in this article's Online Repository at www.jacionline.org). We hand searched the bibliographies of included studies and contacted experts in the field for relevant studies. One additional study was unpublished at the time of the search and coauthored by the reviewers.^19^

### Study selection criteria

Peer-reviewed published studies in any language from any year were eligible for inclusion. Studies were required to be population based, with an average age of participants of greater than 18 years. Studies could be cohort, case–control, or cross-sectional designs. The exposure of interest was atopic eczema (atopic dermatitis). The comparator was person years without atopic eczema. Outcomes were major cardiovascular outcomes: angina, myocardial infarction, coronary revascularization, heart failure, cardiac arrhythmias, stroke, and cardiovascular death. Studies of localized or other types of eczema, such as hand eczema and seborrheic or contact dermatitis, were not eligible for inclusion.

### Data extraction and quality assessment

We used an online literature-reviewing data management program (Covidence) to facilitate collaboration and data extraction between reviewers. Two reviewers (A.A. and A.M.Y.) independently screened titles and abstracts in duplicate unblinded. Full-text articles were retrieved where studies fulfilled inclusion criteria or where there was ambiguity. Disagreement was resolved through discussion with a third reviewer (S.M.L.), where necessary. A.A. and A.M.Y. extracted data independently and in duplicate to minimize bias and errors using a standardized data extraction tool that was piloted on the first 3 eligible texts.

Additional information was requested from authors by e-mail, where needed. A full list of data extracted can be found in the supplementary material in this article's Online Repository at www.jacionline.org. Principal summary measures included odds ratios (ORs), hazard ratios (HRs), and incidence rate ratios (IRRs; all measures of relative risk [RR]).

We grouped studies by the cardiovascular outcome under study and synthesized them narratively. All studies were of sufficient quality for inclusion in the narrative synthesis. We only considered information on the interaction between atopic eczema and covariates if it was formally assessed in the original publication. Study outcomes considered sufficiently homogeneous and without a high risk of bias were eligible for inclusion in meta-analyses to obtain pooled effect estimates.

We compared RR estimates for the relationship between atopic eczema and cardiovascular outcomes between studies. We used estimates that were adjusted for confounders, where available. If fully adjusted estimates were unavailable for a particular study, we used minimally adjusted (age and sex) estimates instead, or if both of these were unavailable, we used fully adjusted estimates that were additionally adjusted for possible mediators (factors on the causal pathway of CVD, such as smoking, body mass index, hyperlipidemia, hypertension, depression, anxiety, diabetes, and/or alcohol). The definition of adjustment differed between studies (see Table E6 in this article's Online Repository at www.jacionline.org).

Risk of bias was assessed by using the Newcastle Ottawa Scale (see Table E4 in this article's Online Repository at www.jacionline.org) and a modified Cochrane Collaboration Grading of Recommendations Assessment, Development and Evaluation (GRADE) tool (see Table E5 in this article's Online Repository at www.jacionline.org).

### Statistical analysis

#### Atopic eczema overall

Associations from longitudinal cohort studies and cross-sectional studies were analyzed separately. For cross-sectional studies, we displayed the extracted ORs of angina, heart failure, myocardial infarction, and stroke (separately) for participants with atopic eczema compared with participants without atopic eczema in forest plots. We used a random-effects meta-analysis model with the inverse variance method of DerSimonian and Laird^20^ to estimate between-study (population) heterogeneity and the I^2^ measure^21^ to quantify the proportion of total variance attributable to this heterogeneity. We present the 95% CI for the pooled estimates, as well as a 95% prediction interval (PI).^22^ Because the CI can convey spurious precision in the presence of population heterogeneity, we interpret the PIs to address our research question. PIs were not calculated for analyses of fewer than 4 studies.

We performed a similar analysis in longitudinal cohort studies, pooling log HRs, log IRRs, or both in the meta-analysis.

#### Atopic eczema severity

All studies assessing the relationship between atopic eczema severity and cardiovascular outcomes were cohorts, with the exception of 2 cross-sectional studies (see Table E3 in this article's Online Repository at www.jacionline.org). We excluded these^10^, ^23^ and displayed the rest in a graph of the (log) HR or IRR against atopic eczema severity (mild, moderate, or severe).

To estimate the relative increase in risk attributable to severity, we constructed a multivariate linear mixed model relating log HRs (IRRs) for each of the 6 outcomes to atopic eczema severity. The linear predictor consisted of an outcome-specific fixed intercept, a random study intercept, an outcome-specific fixed slope for severity (assuming linearity), and random error. We performed a Bayesian analysis on this model with uninformative “priors” (assumptions about the value of unknown parameters that affect estimation), except for the precision of the observed log HRs (IRRs), which we gave the informative priors of the inverse of their observed variance. We report the means and 2.5 and 97.5 quantiles of the posterior distributions, which are the pooled estimates, and their 95% credibility intervals (CrI; analogous to the 95% CI for the mean). We used a Bayesian instead of a frequentist method because correct inference can be achieved with smaller samples than are necessary for frequentist mixed-effects models.^24^

We performed a secondary analysis to estimate the generalized increase in risk attributable to atopic eczema severity. Using a similar model, we changed the fixed outcome intercept to a random intercept and the fixed outcome slopes to a single fixed slope with a random slope effect. The variance of the random slope estimates the between-outcome variability in risk increase and can be used to estimate the distribution of risk increase across all cardiovascular outcomes. In addition to the measures above, we report the 95% uncertainty interval, which is analogous to the 95% PI, by summing the slope and its estimated random effect and reporting the 2.5 and 97.5 quantiles of this posterior distribution. The code for both models is available in the supplementary material in this article's Online Repository at www.jacionline.org. All statistical analyses were performed in R software (version 3.4.3)^25^ by using the “meta”^26^ and “rjags”^27^ packages. No subgroup analyses were undertaken.

## Results

**Fig 1:**
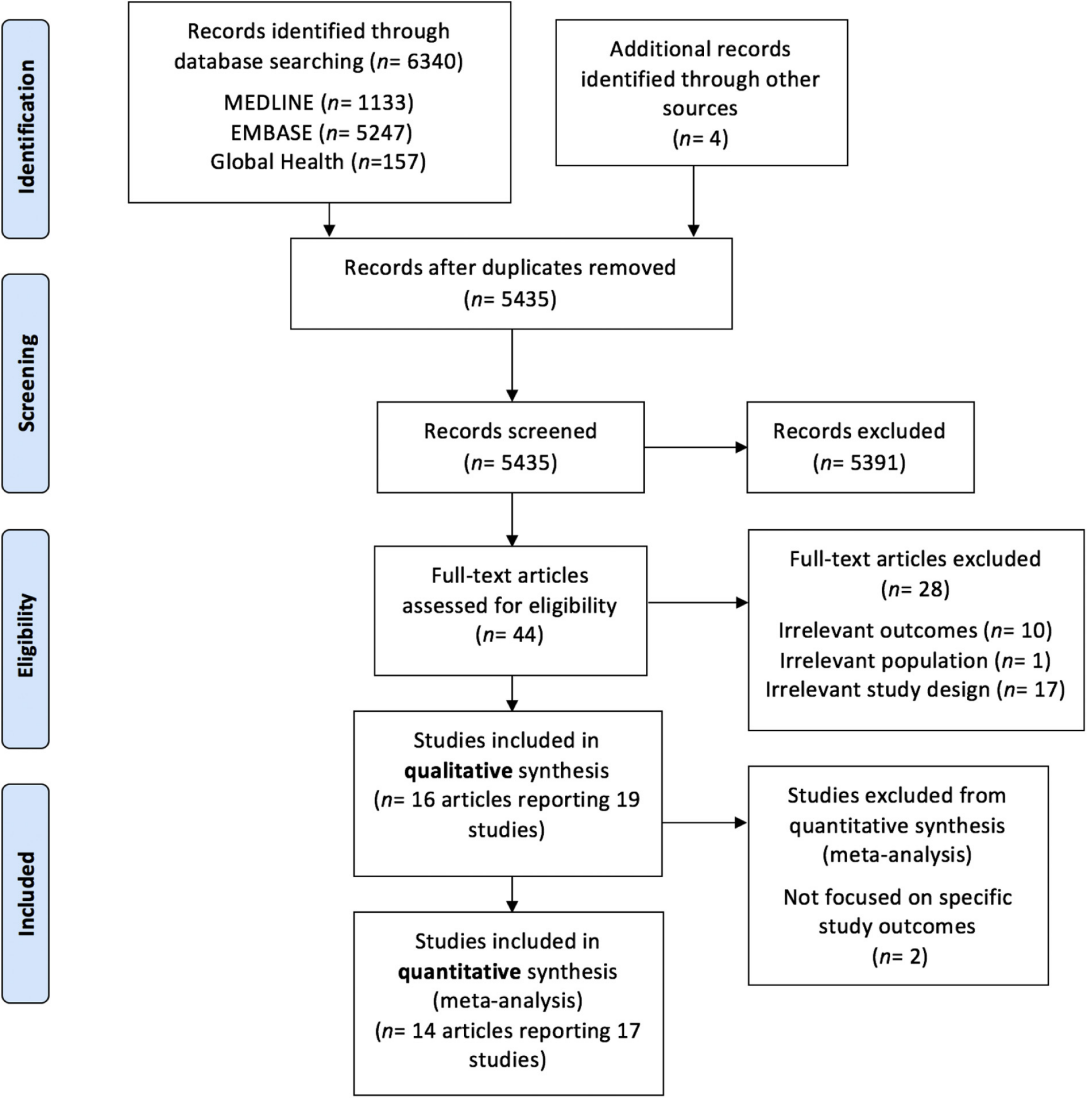
PRISMA flow chart: results of the search strategy.

We screened 5435 records after deduplication, assessing 44 full texts and finding 16 relevant articles reporting 19 different studies, 17 of which were synthesized in the meta-analyses. All relevant studies were available in English. See Fig 1 for the PRISMA flow chart detailing the process of study selection, including reasons for exclusion.

### Study characteristics

Table E1 in this article's Online Repository at www.jacionline.org details the characteristics of studies matching eligibility criteria. Eight studies were longitudinal cohort studies,^10^, ^11^, ^12^, ^19^, ^28^, ^29^, ^30^, ^31^ 1 was a very restricted cohort study described as a case-control study,^32^ and 10 were cross-sectional studies (reported in 8 articles).^10^, ^23^, ^33^, ^34^, ^35^, ^36^, ^37^, ^38^ Population sizes in individual studies ranged from 4,970 subjects to 72,651,487 total population. Stroke and myocardial infarction were the most common cardiovascular outcomes under study. Seven studies assessed the risk of cardiovascular outcomes by atopic eczema severity, with 6 of these studies defining atopic eczema severity by use of systemic treatments^10^, ^12^, ^19^, ^23^, ^28^ and 1 study using number of dermatology visits as a proxy for severity.^11^ Data for our other secondary objectives, including effect modification by sex or the possible role of treatments in cardiovascular risk, were largely unavailable.

### Risk of bias within studies

Tables E4 and E5 provide details of study quality based on the Newcastle Ottawa Scale and GRADE. Our GRADE assessments of each outcome were of either low or very low quality of evidence to support these associations. Although no outcomes were at high risk of bias, the association estimates, definitions of exposure, outcomes, and populations were heterogeneous, and CIs were wide.

### Results of individual studies

Table E2 in this article's Online Repository at www.jacionline.org details the main reported results of included studies. Two studies were excluded from the quantitative meta-analysis because their outcomes under study were not specifically focused. Marshall et al^32^ found no significant association between atopic eczema and their composite outcome of several CVDs (OR, 1.032; 95% CI, 0.744-1.432; *P* = .8489), whereas Radtke et al^36^ found a significantly lower unadjusted prevalence ratio of ischemic heart disease (OR, 0.83; 95% CI, 0.80-0.86) in those with atopic eczema compared with those without.

### Results of meta-analyses

#### Association between atopic eczema and the risk of CVD outcomes

##### Cross-sectional studies

**Fig 2:**
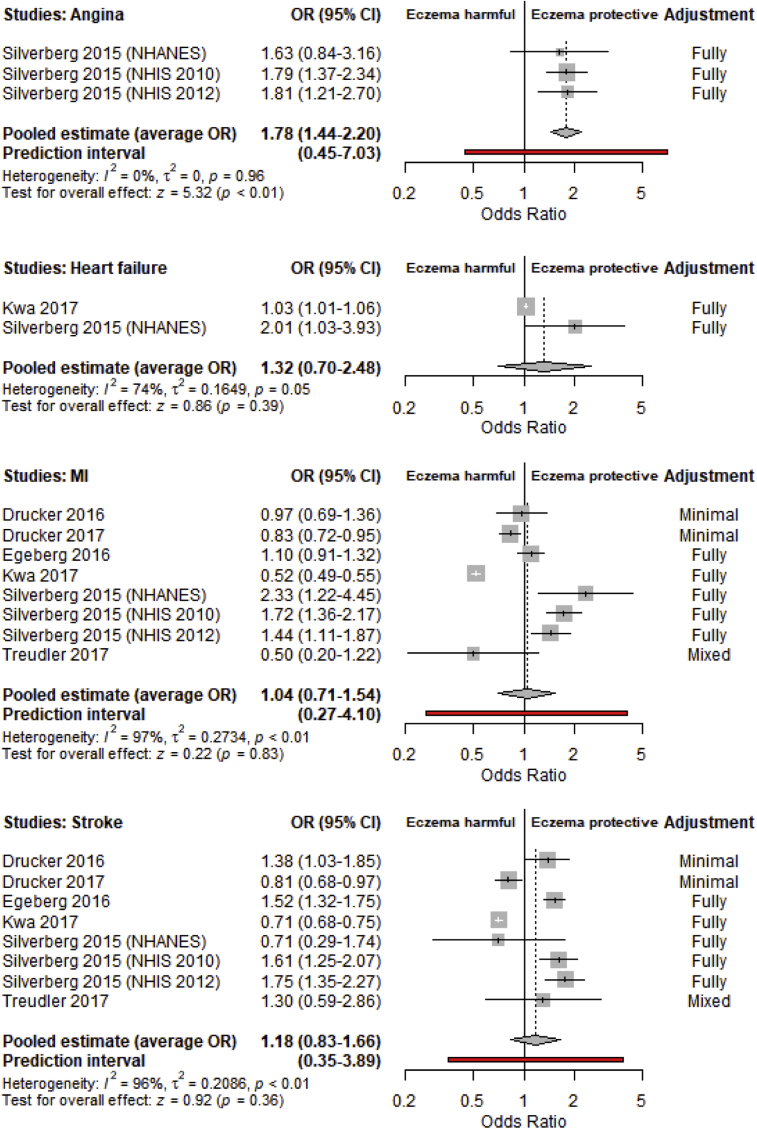
Forest plots of cross-sectional studies estimating the association between atopic eczema and angina, heart failure, myocardial infarction, and stroke. Box sizes are proportional to study weighting in the random-effects meta-analysis, where larger boxes indicate greater precision. *NHANES*, National Health and Nutrition Examination Survey; *NHIS*, National Health Interview Survey.

Three cross-sectional studies estimated the association between atopic eczema overall and angina, with an average OR of 1.78 (95% CI, 1.44-2.20) and 95% PI of 0.45 to 7.03. For heart failure, the average OR between 2 studies was 1.32 (95% CI, 0.70-2.48). The 95% PI was not estimated because there were too few studies. Eight cross-sectional studies provided estimates for the association between atopic eczema and each of myocardial infarction and stroke. For both outcomes, there was substantial heterogeneity between studies (I^2^, 97% and 96%, respectively) and no evidence that the average OR was different from 1, with wide PIs (myocardial infarction: OR, 1.04; 95% PI, 0.27-4.10; stroke: OR, 1.18; 95% PI, 0.35-3.89). Wide PIs for all outcomes from cross-sectional studies suggested a wide range of population effects, some of which might associate having atopic eczema with lower odds and some with higher odds of having a myocardial infarction or stroke; however, cohort studies were more homogeneous than cross-sectional studies and had more consistent estimates. All forest plots for cross-sectional studies are shown in Fig 2.

##### Longitudinal cohort studies

**Fig 3:**
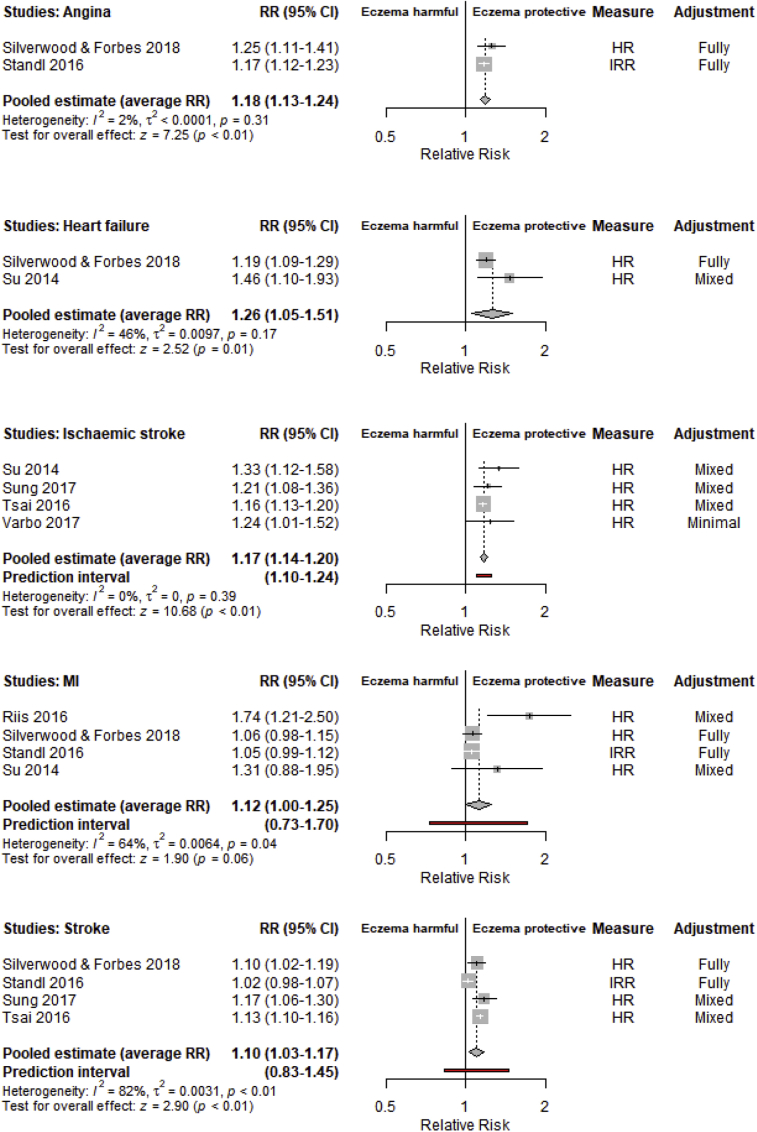
Forest plots of longitudinal cohort studies estimating the association between atopic eczema and the risk of angina, heart failure, myocardial infarction, stroke, or ischemic stroke. Box sizes are proportional to study weighting in the random-effects meta-analysis, where larger boxes indicate greater precision.

Cohort studies were of more interest for their ability to demonstrate temporality. Forest plots for cohort studies are shown in Fig 3. Few studies provided estimates for the effect of atopic eczema on the risk of angina, heart failure, and cardiovascular death. The average RR for angina (n = 2) was estimated to be 1.18 (95% CI, 1.13-1.24), and that for heart failure (n = 2) was estimated to be 1.26 (95% CI, 1.05-1.51). PIs were not estimable. The only study to report an effect of atopic eczema on cardiovascular death^19^ provided no evidence of an association (HR, 0.98; 95% CI, 0.91-1.05).

Four longitudinal cohort studies provided estimates for the effect of atopic eczema and each of the risk of stroke, ischemic stroke, and myocardial infarction. There was evidence that both the average effect and the distribution of population effects supported an increased risk of ischemic stroke (average RR, 1.17; 95% CI, 1.14-1.20; 95% PI, 1.10-1.24). For myocardial infarction and stroke, there was evidence that the average association was an increase in risk but not across the entire population distribution, as indicated by 95% CIs of greater than 1 but 95% PIs that spanned 1 (myocardial infarction: average RR, 1.12; 95% CI, 1.00-1.25; 95% PI, 0.73-1.70; stroke: average RR, 1.10; 95% CI, 1.03-1.17; 95% PI, 0.83-1.45).

#### Association between atopic eczema severity and risk of CVD outcomes

**Fig 4:**
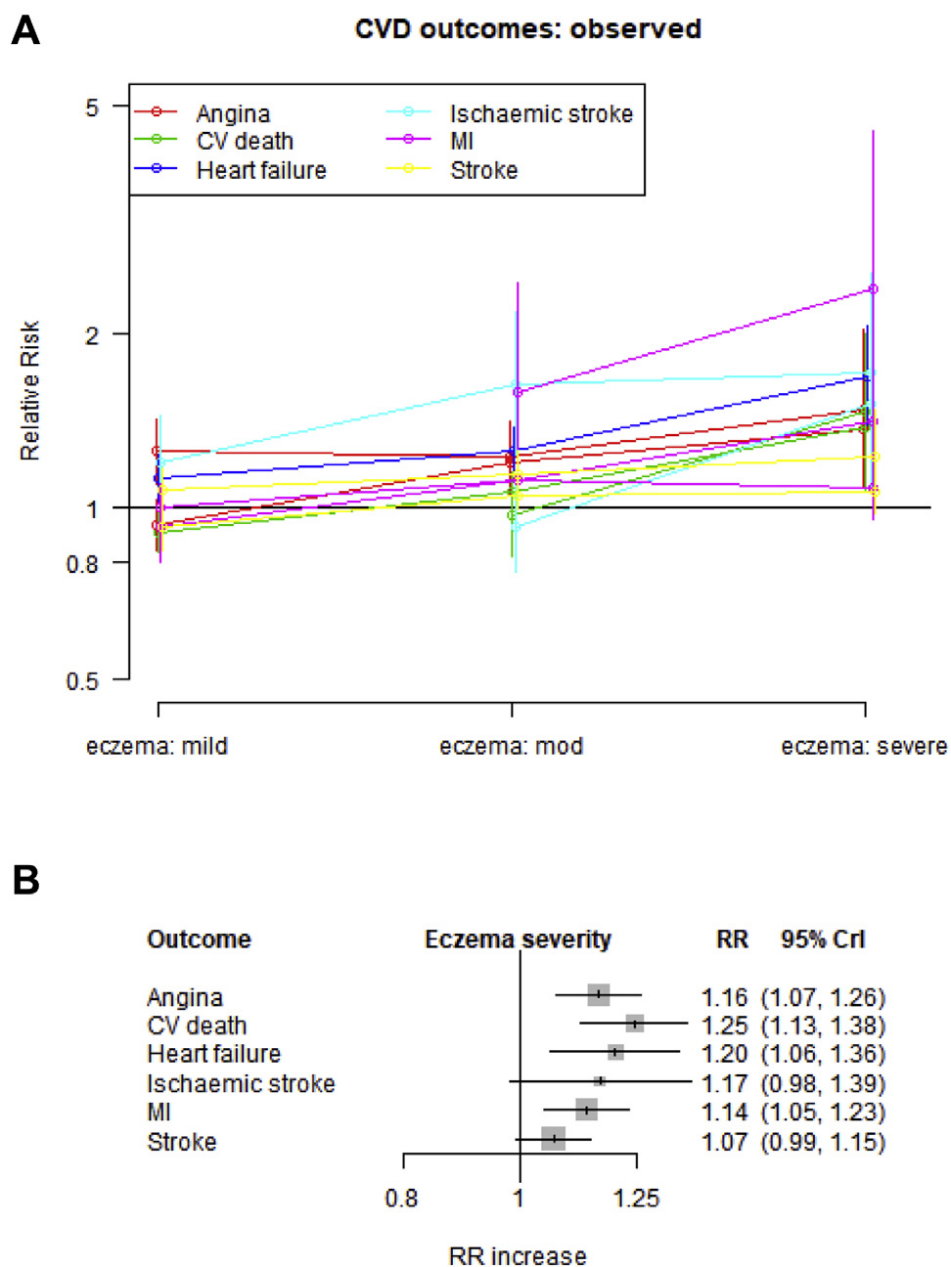
RR of cardiovascular outcomes (compared with subjects without atopic eczema) plotted against atopic eczema severity. **A,** Observed associations with CIs in each study and lines connecting within-study estimates. **B,** Estimated relative increase in risk (ie, slope) for each outcome with 95% CrIs. *CV*, Cardiovascular; *MI*, myocardial infarction.

The risk of having all 6 outcomes appeared to increase with increasing atopic eczema severity, and the slope of this relationship appeared very similar between studies and outcomes (Fig 4, *A*). All 6 slopes estimated from the multivariate Bayesian model were positive, supporting this association (Fig 4, *B*).

Atopic eczema severity was associated with an increased risk of angina (n = 2; RR per-unit increase in severity, 1.16; 95% CrI, 1.07-1.26), heart failure (n = 1; RR, 1.20; 95% CrI, 1.06-1.36), cardiovascular death (n = 2; RR, 1.25; 95% CrI, 1.13-1.38), and myocardial infarction (n = 3; RR, 1.14; 95% CrI, 1.05-1.23) in patients with atopic eczema. There was no statistically significant evidence for the association with stroke (n = 2; RR, 1.07; 95% CrI, 0.99-1.15) or ischemic stroke (n = 2; RR, 1.17; 95% CrI, 0.98-1.39).

The mean RR increase in having any of the prespecified cardiovascular outcomes was 1.15 (95% CrI, 1.09-1.21) with increasing severity categories (eg, mild to moderate or moderate to severe). The uncertainty interval within which we expect to find 95% of all RRs was 1.04 to 1.28, providing evidence for an association in patients with atopic eczema between severity of disease and an increased risk of CVD.

We have not formally explored causes of heterogeneity because the low number of studies for these outcomes provides little power to detect them. We could not explore publication bias with funnel plots because in the presence of high heterogeneity, there is no reason to expect a plot of estimates against their SEs to have a funnel shape.^39^

## Discussion

Our systematic review and meta-analysis found an association with stroke and myocardial infarction in cohort studies and an association with increasing disease severity across all cardiovascular outcomes under study. High heterogeneity and imprecision resulted in low quality of evidence to support these associations; however, all outcomes were judged to be of low risk of bias.

### Strengths and limitations

The strengths of our study include our preregistered and published protocol, increasing the transparency of our study. A librarian and medical professional coplanned a systematic and broad search strategy attempting to identify all studies meeting eligibility criteria. Two authors (A.A. and A.M.Y.) independently assessed search results by using an online data management program to minimize bias and errors in screening, data extraction, and quality assessment of studies. This is the first systematic review and meta-analysis (to our knowledge) to review the association between CVD and atopic eczema severity. We directly addressed uncertainties in observational data by calculating credibility and PIs where enough studies were returned for the association between atopic eczema and cardiovascular outcomes. PRISMA guidelines informed the reporting of this systematic review.^40^

It is still possible that relevant articles might have been missed, despite an extensive search. Some of our conclusions are limited by a lack of systematic reporting of outcomes, heterogeneity in the definitions of atopic eczema and atopic eczema severity, and adjustment models used in included studies. The statistical methods address some of these limitations with the calculation of PIs. Some outcomes with 2 studies could not estimate a PI, and those with 3 or 4 studies had a not well-estimated PI; however, the results for outcomes with more data available were robust. Limited sensitivity analyses could be undertaken because of the small of numbers of studies returned, and some of the questions we set out to answer could not be answered because of an absence of evidence. Between-study heterogeneity and corresponding statistics are not well estimated from a small number of studies, which is reflected in our GRADE assessments of low-quality evidence, making it difficult to draw firm conclusions from this review.^41^ Publication bias could not be formally assessed because of the presence of high heterogeneity and small study numbers.

### Comparison with previous studies

A previous systematic review reported no significant association between atopic eczema and myocardial infarction and stroke.^16^ However, this review pooled crude estimates preferentially, meta-analyzed the associations from cross-sectional and longitudinal cohort studies together, and included fewer relevant studies. In addition, this previous review did not address atopic eczema severity.

Although individual studies have come to varied conclusions on the association between atopic eczema and cardiovascular outcomes, systematic reviews have contributed to the evidence that atopic eczema is associated with an increased prevalence of certain mediators on the causal pathway to CVD. Zhang et al^14^ found an increased prevalence of obesity in North America and Asia, whereas smoking,^42^ depression, anxiety, hyperlipidemia, and hypertension have all been associated with atopic eczema. Meta-analyses have not shown evidence for increased alcohol use^43^ or type 2 diabetes.^16^ Other atopic diseases, such as asthma, have also been linked to increased cardiovascular outcomes.^44^ Silverwood et al^19^ adjusted for mediators, including increased body mass index, smoking, severe alcohol use, hyperlipidemia, hypertension, diabetes, depression, and anxiety, and still found a positive association between unstable angina and heart failure and atopic eczema (overall). Standl et al^10^ found no association between a number of genetic risk factors for CVD and atopic eczema. Alternative explanations contributing to an increase in risk factors could be behavioral factors linked to the burden of disease, such as reduction of physical activity caused by discomfort, and the established link between inflammation and CVD.

### Our results in context

Although we found no association overall between atopic eczema and cardiovascular outcomes, the association between increasing atopic eczema severity and cardiovascular outcomes appeared consistent across the small number of included studies. Further studies would have been desirable. The determination of absolute risks was beyond the scope of this review, but Silverwood et al^19^ did highlight that absolute risks can remain low, even in the context of high RRs.

The question remains whether there is a causal link between atopic eczema and CVD. The link between psoriasis and CVD is now established; however, the 2 diseases have distinct immunoprofiles, with a T_H_2 signal predominating in patients with psoriasis and T_H_1 signal persisting in patients with chronic atopic eczema.^45^ Factors supporting a causal link between atopic eczema and cardiovascular outcomes include mechanistic theories linking inflammation and CVD, the evidence for temporality, and the evidence for a dose-response relationship between atopic eczema severity and cardiovascular outcomes.

Challenges remain because of the types of data used to answer this question. Only 1 study defined atopic eczema severity as a time-updated variable.^19^ Therefore there might have been misclassification of severity, leading to overestimating or underestimating the risk associated with mild or moderate disease. In addition, childhood-onset compared with adult-onset atopic eczema was poorly reported in the literature but could potentially be a significant issue. Misclassification bias could account for an increased risk of cardiovascular outcomes in patients with atopic eczema because those with severe disease are often defined by receiving systemic therapies, although for most of the outcomes studies, this is not likely to be a particular issue because we have focused on major cardiovascular outcomes that are likely to present for care.

The study by Sung et al^29^ was the only study in this review to disregard treatment while defining atopic eczema severity by using number of dermatology visits as a proxy for severity. A dose response remained between increasing numbers of dermatologic visits and increasing risk of ischemic stroke. Increased health care consumption could increase the likelihood of picking up alternative diagnoses, such as atrial fibrillation; however, this is unlikely to account for all of the associations seen.

Silverwood et al^19^ undertook sensitivity analyses adjusting for high-dose oral glucocorticoid use by removing patients treated with cyclosporine or methotrexate; however, none of these approaches significantly affected their finding that moderate-to-severe atopic eczema was associated with an increased risk of specific CVD outcomes, suggesting that the association persisted independent of systemic treatment type. It is possible that some systemic therapies can protect against CVD. In patients with psoriasis, methotrexate therapy might be protective against cardiovascular outcomes; however, glucocorticoids and cyclosporine could increase cardiovascular risk through hypertension and hyperlipidemia.

It would be beneficial for future studies to separate confounders and then subsequently adjust for both confounders and mediators to address the important question of whether the inflammatory burden of atopic eczema contributes to CVD independently. This would aid delineation of the contribution of increased burden of cardiovascular risk factors and increased risk of CVD conferred by the disease itself.

### Conclusion

This study is the first to meta-analyze the association between increasing atopic eczema disease severity and cardiovascular outcomes, demonstrating evidence of a dose-response relationship between increasing severity and cardiovascular outcomes. Further studies are required to understand the basis of this finding to support targeted prevention strategies for those with moderate-to-severe atopic eczema.

**Clinical implications**

Understanding the basis of the dose-response relationship between atopic eczema disease severity and cardiovascular outcomes is key to supporting targeted prevention strategies for those with moderate-to-severe atopic eczema.

## Supplementary data

Online repository text
