# Supplementary material for: Atopic eczema and major cardiovascular outcomes: A systematic review and meta-analysis of population-based studies
Source: J Allergy Clin Immunol. 2019 May;143(5):1821–9. doi: 10.1016/j.jaci.2018.11.030 (PMC6497108; doi:10.1016/j.jaci.2018.11.030)
Supplement: Online repository text [file mmc1.docx]

**Supplementary material**

**Atopic eczema and major cardiovascular outcomes: a systematic review and meta-analysis of population-based studies**

Anna Ascott*, Amy Mulick*, Ashley M Yu, David Prieto-Merino, Morten Schmidt, Katrina Abuabara, Liam Smeeth, Amanda Roberts, Sinéad M Langan

*Joint first authors

**Contents:**

1. **Ovid MEDLINE search strategy**
2. **Changes to the original protocol**
3. **Extracted data items**
4. **Results of search- studies matching eligibility criteria (Table S1)**
5. **Results of studies matching eligibility criteria (Table S2)**
6. **Study definitions of atopic eczema severity (Table S3)**
7. **Risk of bias in individual studies using the Newcastle Ottawa Scale (NOS) (Table S4)**
8. **Reasons to up- or down-grade quality of evidence in Grading of Recommendations, Assessment, Development and Evaluation (GRADE)**
9. **GRADE assessments and explanation (Table S5)**
10. **Detail of models of adjustment for studies combined in the meta-analyses (Table S6)**
11. **Detail on statistical methods**
12. **Detail on statistical results**
13. **Bayesian model for estimating atopic eczema severity and the risk of six cardiovascular outcomes**
14. **Bayesian model for estimating atopic eczema severity and the average risk for cardiovascular outcomes**
15. **Ovid MEDLINE search strategy**

1. Dermatitis, Atopic/

2. exp Eczema/

3. (eczem* or atopic dermatit*).mp. [mp=title, abstract, original title, name of substance word, subject heading word, keyword heading word, protocol supplementary concept word, rare disease supplementary concept word, unique identifier, synonyms]

4. or/1-3

5. intracranial embolism/

6. exp Intracranial Hemorrhages/

7. exp Intracranial Arterial Diseases/

8. exp Brain Ischemia/

9. (intracranial embolism* or intracranial h?emorrhage* or intracranial arterial disease* or intracranial thrombos* or stroke* or cerebrovascular accident* or cerebrovascular diseas* or cva or cerebral artery diseas* or brain isch* or brain infarct* or brain h?emorrhag* or occlusive cerebrovascular disease*).mp. [mp=title, abstract, original title, name of substance word, subject heading word, keyword heading word, protocol supplementary concept word, rare disease supplementary concept word, unique identifier, synonyms]

10. exp Cardiovascular Diseases/

11. (myocardial isch?emia* or heart arrest* or heart attack* or myocardial infarct* or acute coronary syndrome* or angina* or isch?emic heart diseas* or coronary arter* or heart fail* or congestive cardiac fail* or ccf or lvf or left ventricular fail* or rvf or right ventricular fail* or heart right ventricle fail* or heart left ventricle fail* or af or cardiovascular diseas* or heart diseas* or vascular diseas* or arrhythmia* or abnormal heart rhythm* or atrial flutter* or heart block* or svt* or supraventricular tachycardia* or bundle branch block*).mp. [mp=title, abstract, original title, name of substance word, subject heading word, keyword heading word, protocol supplementary concept word, rare disease supplementary concept word, unique identifier, synonyms]

12. exp Myocardial Revascularization/

13. exp Percutaneous Coronary Intervention/

14. (myocardial revasculari?ation* or percutaneous coronary intervention* or heart muscle revasculari?ation* or coronary revasculari?ation* or percutaneous transluminal angioplast* or coronary artery obstruction* or transluminal coronary angioplast* or coronary artery surger* or interventional cardiovascular procedure*).mp. [mp=title, abstract, original title, name of substance word, subject heading word, keyword heading word, protocol supplementary concept word, rare disease supplementary concept word, unique identifier, synonyms]

15. (cardiac arrest* or heart death* or card* death* or (cardiovascular adj3 mortalit*)).mp. [mp=title, abstract, original title, name of substance word, subject heading word, keyword heading word, protocol supplementary concept word, rare disease supplementary concept word, unique identifier, synonyms]

16. or/5-15

17. 4 and 16

1. **Changes to the original protocol**

IIa) In our published protocol we stated that we would search to April 2017, however, we knew of relevant studies that had since been published and therefore updated our search to December 2017.

IIb) If we observed high heterogeneity between studies, we planned to explore reasons for this in sensitivity analyses. However, few studies were returned, providing little power for detection.

IIc) We planned to look for publication bias using standard approaches including funnel plots and Egger tests, however, after consulting with an experienced statistician, in the presence of high heterogeneity there is no reason to expect a plot of estimates against their standard errors to have a funnel shape.

1. **Extracted data items**

IIIa) study details: author information, publication year, design, sponsorship, geographical location, healthcare setting, length of follow-up time if relevant, sampling and recruitment methods, period of study, aims and objectives;

IIIb) population characteristics: population size, mean and median age, gender distribution, inclusion and exclusion criteria;

IIIc) exposure: definition of atopic eczema as an exposure, number of exposed subjects, details of atopic eczema severity and treatment, age at onset of atopic eczema if available;

IIId) comparators: definition of unexposed subjects, number of comparators;

IIIe) outcomes: definition and identification of cardiovascular outcomes (angina, myocardial infarction, coronary revascularisation, heart failure, cardiac arrhythmias, stroke and cardiovascular death), number of subjects with the outcome.

1. **Supplementary table S1. Results of search- studies matching eligibility criteria**

| **First author, publication year** | **Research design** | **Setting** | **Study period** | **Population in detail** | **Exposure definition and ascertainment** | **Outcome definition and ascertainment** | **Outcome type** | | | | | |
| --- | --- | --- | --- | --- | --- | --- | --- | --- | --- | --- | --- | --- |
|  |  |  |  |  |  |  | **Angina** | **MI** | **HF** | **Stroke** | **Cardiovascular death** | **Other** |
| **Andersen, 2016^1^** | Cohort | Danish National Patient Register, Denmark | 1997-2012 | Danish population above ≧15 yrs | At least one inpatient or outpatient hospital diagnosis of ICD-8* 691 or ICD-10** L20 | ICD-8 codes 1978-1994, ICD-10 codes from 1994 to present. I21-I22, I63-I64, and cardiovascular death (I00-I99) |  | X |  | X (ischaemic stroke) | X | MACEs (composite endpoint of MI, ischemic stroke, CV death) |
| **Drucker, 2016^2^** | Cross-sectional | Nurses’ Health Study 2 (NHS2), USA | 1989-2009 | Female registered nurses aged 25-49 in 1 of 14 states | Self-reported- 'Yes' when asked whether they 'ever had clinician diagnosed eczema (atopic dermatitis)?' | Self-reported then confirmed by medical records. Otherwise, information was confirmed by interview or letter. If this was deemed to be 'probable', they were included |  | X |  | X |  |  |
| **Drucker, 2017^3^** | Cross-sectional | Canadian Partnership for Tomorrow Project, Canada | 2009 onwards | Any resident 30-74 years attending for assessment at the project centre; random selection from population-based data | Self-reported- ‘Yes’ to ‘Has a doctor ever told you that you had any of the following conditions: eczema?’ | Self-reported ‘yes’ to ‘Has a doctor ever told you that you had any of the following conditions (MI/stroke)? If yes, please provide your age when you were first diagnosed’ |  | X |  | X |  |  |
| **Egeberg et al, 2016^4^** | Cross-sectional | Danish National Patient Register, Denmark | 1995-2012 | Danish citizens ≧18 yrs | At least one inpatient or outpatient diagnosis of ICD-10** L20 | ‘Administration codes’ |  | X |  | X |  |  |
| **Kwa, 2017^5^** | Cross-sectional | National  Inpatient Sample (NIS), USA | 2002–2012 | US inpatients ≧18 years old in NIS | A primary and/or secondary diagnosis of ICD-9-CM*** codes 691.8 and 692.9 | Data pre-coded by Association for Healthcare Research and Quality (AHRQ) |  | X | X | X |  |  |
| **Marshall, 2013^6^** | Very restricted cohort study (but described and analysed as case control) | MarketScan^TM^  Commercial Claims and Encounters database | 2005–2007 | Patients with Insurance database- MarketScan Commercial Claims and Encounters | At least two clinical diagnoses (inpatient or outpatient) of ICD-9-CM*** 691.XX except 691.0X | Two diagnosis codes or a diagnosis code plus a  cardiovascular-related procedure code (ICD-9-CM*** 410–414, 428, 433, 434, 435) |  |  |  |  |  | IHD, TIA, HF,  occlusion and stenosis of pre-cerebral arteries, and occlusion of cerebral arteries |
| **Radtke, 2017^7^** | Cross-sectional | Gmuender Ersatzkasse (GEK) (national health insurance database), Germany | 2009 | German citizens ≧18 yrs insured with GEK | At least one ambulatory, hospital or disability related diagnosis of ICD-10** L.20 in 2009 | Clinically diagnosed and defined by ICD-10** codes |  |  |  |  |  | Ischaemic heart disease |
| **Riis, 2016^8^** | Cohort | Danish National Patient Register, Denmark | 1977-2013 | Danish citizens born between 1947 to 1983 | At least two inpatient or outpatient hospital diagnoses of ICD-8* 691 or ICD-10** L20 | Clinically diagnosed and defined by ICD codes (ICD-8 410, ICD-10 I21) |  | X |  |  |  |  |
| **Silverberg, 2015^9^** | Cross-sectional | National Health and Nutrition Examination Survey (NHANES), USA | 2005-2006 | Patients ≥20 years old, non-institutionalized adults nationally and in each state | Self-reported- ‘Yes’ to 'Have you ever had an itchy rash that was coming and going for at least 6 months?', 'Have you had this itchy rash at any time in the past 12 months?', 'Has this rash ever affected the folds of the elbows, behind knees, front ankles, under buttocks, around the neck ears or eyes?' | Self-reported ‘Yes’ to 'Has a doctor or other health professional ever told you that you had a heart attack (also called myocardial infarction) or angina (angina pectoris), or congestive heart failure or stroke?' | X | X | X | X |  |  |
|  | Cross-sectional | National Health Interview Survey (NHIS), USA | 2010 | ≥18 years old who participated in population based survey | Self-reported- ‘Yes’ to 'During the past 12 months have you had dermatitis, eczema or any other red, inflamed rash?' | As above | X | X |  | X |  |  |
|  | Cross-sectional | National Health Interview Survey (NHIS), USA | 2012 | Same as above | Self-reported- ‘Yes’ to 'During the past 12 months have you been told by doctor/other healthcare professional you have eczema or any kind of skin allergy' | As above | X | X |  | X |  |  |
| **Silverwood and Forbes, 2018^10^** | Cohort | Clinical Practice Research Datalink (CPRD), UK | 1998-2015 | ≥18 years old, with previously collected routine health data present in CPRD | Primary diagnosis using ICD-10** codes in CPRD (using Read codes) and Hospital Episode Statistics (HES) and two atopic eczema treatments on separate dates | Primary diagnosis in fields of any episode in CPRD or HES, confirmed with ICD-9 or ICD-10 code, and procedures in OPCS classification (interventions and procedures data), | X (unstable angina) | X | X | X | X | Coronary revascularisation |
| **Standl, 2016^11^** | Cross-sectional | AOK PLUS Saxony (national health insurance database), Germany | 2012-2014 | Patients ≥40 yrs who were consistently insured (or died) from 2005-2014, with data in the German National Health Insurance AOK Plus database | At least two outpatient hospital diagnoses of ICD-10** L20 | ICD-10 codes | X | X |  | X |  |  |
|  | Cohort | AOK PLUS Saxony (national health insurance database), Germany | Diagnosis: 2005/2008-Outcome assessment: 2008-2014 | Same as above | At least two outpatient hospital diagnoses of ICD-10** L20 | As above | X | X |  | X |  |  |
| **Su, 2014^12^** | Cohort | National Health Insurance database, Taiwan | 2005-2009 | General Taiwanese population ≥20 yrs | New diagnosis of ICD-9-CM 691.X | ICD 9 codes 436,433.x,434.x, or 437.1x, and physician diagnosed with CT/MRI evidence within 14 days |  | X | X | X |  |  |
| **Sung, 2017^13^** | Cohort | Longitudinal Health Insurance  Database, Taiwan | 2000-2010 | General Taiwanese population | New inpatient or outpatient diagnosis of ICD-9-CM 691 with at least three consensus diagnoses | ICD-9 codes 430-432 and clinical diagnosis |  |  |  | X (all stroke, ischaemic, and haemorrhagic) |  |  |
| **Treudler, 2017^14^** | Cross-sectional | Germany, LIFE-Adult-Study (Leipzig Research Centre for Civilization Diseases), Germany | 2011-2014 | Random sample of residents of the city of Leipzig, Germany, with a target age range 40-79 yrs and a small group of 18–39 years old (age- and gender-stratified) | Self-reported physician diagnosed atopic dermatitis (ever) | Self-reported physician diagnosed |  | X |  | X |  |  |
| **Tsai, 2016^15^** | Cohort | National Health Insurance database, Taiwan | 2000-2011 | General Taiwanese population | New diagnosis (at least two medical visits) with ICD-9-CM*** 690.X, 691.X, and 692.X | ICD-9-CM codes: 430–438 |  |  |  | X (all stroke and ischaemic stroke) |  | X |
| **Varbo, 2017^16^** | Cohort | The Copenhagen General Population Study, Denmark | 2003-2014 | Randomly selected members of the general population in Copenhagen, Denmark; reflect adults 20-100+ years | Self-reported- ‘Yes’ to 'Does any type of food, medicine, grass, flowers, animal-hairs, or anything else give you eczema?' | ICD codes (ICD8: 431-438, ICD 10: I60-I69 + G45). For cerebrovascular disease, hospital records were requested and diagnosis of ischemic stroke was validated by two independent and blinded doctors. |  |  |  | X (ischaemic stroke) |  |  |

*Table Legend:* *ICD-8 (International Classification of Diseases- Revision 8). 691- Infantile eczema and related conditions. **ICD-10 (International Statistical Classification of Diseases and Related Health Problems). I21- acute myocardial infarction, I22- subsequent myocardial infarction, I63- cerebral infarction, I64- stroke, not specified as haemorrhage of infarction, L20- atopic dermatitis. ***ICD-9-CM (International Classification of Diseases, Ninth Revision, Clinical Modification). 410–414- ischemic heart disease, 428- heart failure, 433- occlusion and stenosis of precerebral arteries, 434- occlusion of cerebral arteries, 435- transient cerebral ischemia, 690- Erythematosquamous dermatosis, 691- topic dermatitis and related conditions, 691.0- diaper or napkin rash, 691.8- Other atopic dermatitis and related conditions, 692- contact dermatitis and other eczema, 692.9- dermatitis NOS. MI- myocardial infarction, HF- heart failure, IHD- ischaemic heart disease, TIA- transient cerebral ischaemia, IRR- incident rate ratios, OR- odds ratio, HR- hazard ratio, RR- risk ratio.

1. **Supplementary table S2. Results of studies matching eligibility criteria**

| **First author, publication year** | **Research design** | **Population size (proportion of males)** | **Follow-up time (years)** | **Subjects with outcome [or exposure for case-control studies] (N, %)** | **Statistical analysis method used** | **Main reported results** | | | | | | **Adjusted for** |
| --- | --- | --- | --- | --- | --- | --- | --- | --- | --- | --- | --- | --- |
|  |  |  |  |  |  | **Angina** | **Myocardial Infarction** | **Heart Failure** | **Stroke** | **Cardiovascular death** | **Other** |  |
| **Andersen, 2016^1^** | Cohort | 174,797 (46.0%) | 15 | MI: 125 (0.435%)  Ischemic stroke: 184 (0.625%)  CV Death: 204 (0.693%) | Poisson regression models, Wald X2 tests |  | Age and sex adjusted IRR: Mild AD 0.82 (0.66-1.02), Severe AD 1.39 (0.95-2.03) |  | Age and sex adjusted IRR: Mild AD 0.92 (0.78-1.11), Severe AD 1.51 (1.08-2.10) | Age and sex adjusted IRR: Mild AD 0.97 (0.82-1.15), Severe AD 1.46 (1.07-2.02) |  | Age, sex, SES, smoking, comorbidities, medication use |
| **Drucker, 2016^2^** | Cross-sectional | 78,702 (0%) |  | Non-fatal MI: 38 (0.480%)  Non-fatal stroke: 52 (0.656%) | Logistic regression |  | Age adjusted OR: 0.97 (0.69-1.36) |  | Age adjusted OR: 1.38 (1.03-1.85) |  |  | Age, ethnicity, BMI, physical activity, smoking, FmHx MI, hormone replacement use, hx HTN, dyslipidaemia, diabetes |
| **Drucker, 2017^3^** | Cross-sectional | 259,119 (38.6%) |  | Non-fatal MI: 210 (0.992%)  Non-fatal stroke: 127 (0.598%) | Multivariable logistical regression |  | Age and sex adjusted OR 0.83 (0.72-0.95) |  | Age and sex adjusted OR 0.81 (0.68-0.97) |  |  | Age, sex, ethnicity, BMI, hx smoking, alcohol, sleep, physical activity, asthma, HTN, diabetes, MI, stroke |
| **Egeberg et al, 2016^4^** | Cross-sectional | 87,307 (38.2% exposed, 8.2% of unexposed) |  |  | Logistic regression, multivariate models for adjusted odds ratios |  | overall: OR 1.1 (0.92-1.33) mild AD: OR 0.88 (0.63-1.23) severe AD: OR 1.23 (0.98-1.54) |  | overall: OR 1.52 (1.32-1.74) mild AD: OR 1.23 (0.94-1.6) severe AD: OR 1.45 (1.19-1.77) |  |  | Age, sex, SES, healthcare consumption (number of dermatology visits) |
| **Kwa, 2017^5^** | Cross-sectional | 72,651,487 |  | MI: 2819 (1.71%)  HF: 13668 (8.29%)  Stroke: 2737 (1.66%) | Survey-weighted binary logistic regression model, propensity score matching models |  | OR 0.56 (0.53-0.58) (hx of MI OR 0.80) | OR 1.10 (1.07-1.13) | OR 0.74 (0.71-0.77) |  |  | Age, sex, ethnicity, mean annual household income, insurance status, number of chronic conditions, hospital region. |
| **Marshall, 2013^6^** | Very restricted cohort study (but described and analysed as case control) | 8,526 (33%) | 1-3 |  | Multivariate logistic regression, main effects model |  |  |  |  |  | IHD, TIA, HF,  occlusion and stenosis of pre-cerebral arteries, and occlusion of cerebral arteries | Healthcare utilization, age, overall health status (Deyo-Charlson comorbidity index), sex, comorbidities, medications used |
| **Radtke, 2017^7^** | Cross-sectional | 1,349,671 |  | Ischemic heart disease: 2725 (5.66%) | Chi-square, prevalence rates |  |  |  |  |  | Ischaemic heart disease |  |
| **Riis, 2016^8^** | Cohort | 53,210 (45%) | 37 | MI: 29 (0.6%) | Cox proportional hazards regression models |  | HR: 1.79 (1.25-2.57) mild AD: 1.62 (1.04-2.51) Severe AD: 2.38 (1.26-4.50) |  |  |  |  | Gender, birth-year, educational level, diabetes, HTN, dyslipidaemia, stroke |
| **Silverberg, 2015^9^** | Cross-sectional- NHANES | 4,970 |  | Angina: 13 (4.6%)  Non-fatal MI: 13 (6%)  HF: 16 (7.7%)  Non-fatal stroke: 6 (2.2%) | Bivariate models-survey-weighted logistic regression, multivariable regression | OR: 1.87 (0.98–3.57) | OR: 2.59 (1.35-4.96) | OR: 2.37 (1.27-4.41) | OR: 0.76 (0.31-1.86) |  |  | BMI, hx smoking, alcohol consumption, vigorous activity in past 30 days, highest level of education, age, household income, ethnicity, sex |
|  | Cross-sectional- NHIS 2010 | 27,157 |  | Angina: 107 (2.1%)  Non-fatal MI: 139 (5.3%)  Non-fatal stroke: 112 (4.3%) | Bivariate models-survey-weighted logistic regression, multivariable regression | OR: 1.87 (1.44-2.42) | OR: 1.73 (1.39-2.16) |  | OR: 1.61 (1.27-2.05) |  |  | BMI, hx smoking, alcohol consumption, vigorous activity in past 30 days, highest level of education, age, household income, ethnicity, sex |
|  | Cross-sectional- NHIS 2012 | 34,525 |  | Angina: 86 (3.2%)  Non-fatal MI: 123 (4.2%)  Non-fatal stroke: 118 (4.2%) | Bivariate models-survey-weighted logistic regression, multivariable regression | OR:1.73 (1.18-2.54) | OR: 1.33 (1.03-1.70) |  | OR: 1.63 (1.27-2.09) |  |  | BMI, hx smoking, alcohol consumption, vigorous activity in past 30 days, highest level of education, age, household income, ethnicity, sex |
| **Silverwood and Forbes, 2018^10^** | Cohort | 1,915,916 (35.6%) | 18 | Unstable angina: 2460 (0.635%)  Non-fatal MI: 5561 (1.435%)  HF: 6441 (1.662%)  Non-fatal stroke: 7149 (1.845%)  CV Death: 10813 (2.791%)  Coronary revascularisation: 5056 (1.305%)  A-fib: 9892 (2.553%) | Cox regression | Unstable angina: Unadjusted: HR= 1.22 (99%CI= 1.14-1.31) Mild: 1.22 (1.11-1.33) Mod: 1.20 (1.08-1.34) Severe: 1.47 (1.10-1.97) | Unadjusted: HR= 1.10 (99%CI= 1.05-1.15) Mild: 1.03 (0.96-1.09) Mod: 1.15 (1.07-1.23) Severe: 1.46 (1.21-1.74) | Unadjusted: HR= 1.21 (99%CI= 1.16-1.26) Mild: 1.12 (1.05-1.19) Mod: 1.27 (1.19-1.35) Severe: 1.71 (1.43-2.05) | Unadjusted: HR= 1.07 (99%CI= 1.03-1.12) Mild: 1.03 (0.98-1.09) Mod: 1.11 (1.04-1.18) Severe: 1.19 (1.00-1.42) | Unadjusted: HR: 1.07 (99%CI= 1.03-1.11) Mild: 0.97 (0.92-1.02) Mod: 1.15 (1.09-1.21) Severe: 1.49 (1.29-1.73) | Coronary revascularisation- Unadjusted: HR= 1.12 (99%CI= 1.07-1.17) Mild: 1.09 (1.02-1.16) Mod: 1.11 (1.04-1.20) Severe: 1.42 (1.17-1.71).  A-fib - Unadjusted: HR= 1.12 (99%CI= 1.08-1.16) Mild: 1.05 (1.00-1.10) Mod: 1.18 (1.12-1.24) Severe: 1.40 (1.21-1.62) | Age at cohort entry, gender, date at cohort entry and practice |
|  |  |  | 18 | Same as above | Cox regression | Adjusted: HR= 1.25 (1.11-1.41) Mild: 1.25 (1.10-1.43) Mod: 1.22 (1.06-1.42) Severe: 1.48 (1.08-2.03) | Adjusted: HR= 1.06 (0.98-1.15) Mild: 1.00 (0.91-1.10) Mod: 1.11 (1.01-1.23) Severe 1.41 (1.15-1.71) | Adjusted: HR= 1.19 (1.10-1.30) Mild: 1.12 (1.02-1.23) Mod: 1.25 (1.14-1.38) Severe: 1.69 (1.38-2.06) | Adjusted: HR= 1.10 (1.02-1.19) Mild: 1.07 (0.98-1.16) Mod: 1.14 (1.04-1.25) Severe: 1.22 (1.01-1.48) | Adjusted: HR= 0.98 (0.92-1.06) Mild: 0.90 (0.84-0.98) Mod: 1.06 (0.98-1.15) Severe: 1.38 (1.17-1.62) | Coronary revascularisation- Adjusted model: HR= 1.14 (1.05-1.24) Mild: 1.12 (1.02-1.23) Mod: 1.14 (1.03-1.26) Severe: 1.47 (1.19- 1.81).  A-fib- Adjusted: HR= 1.11 (1.04-1.18) Mild: 1.05 (0.97-1.13) Mod: 1.17 (1.08-1.27) Severe: 1.38 (1.17-1.62) | Current calendar period, time since diagnosis, IMD at cohort entry, time-varying asthma |
| **Standl, 2016^11^** | Cross-sectional | 1,180,678 (44.59% of exposed, 36.24% of unexposed) |  | Angina: 1753 (4.79%)  MI: 651 (1.78%)  Non-fatal stroke): 1581 (4.32%) | Linear regression | Age and sex adjusted: RR= 1.18 (1.13-1.23) p=4.31x10^-12 mild:1.20 (1.10-1.31) p=0.0000373 Mod:1.33 (1.25-1.42) p=<0.0001 Severe: 1.6 (1.45-1.77) p=<0.0001 | Age and sex adjusted: RR= 0.98 (0.90-1.05) p=0.542 mild AD: 0.94 (0.82-1.08) Moderate AD: 0.95 (0.85-1.05) Severe AD: 1.12 (0.94-1.32) |  | Age and sex adjusted: RR= 1.06 (1.01-1.11) p=0.027 Mild: 1.03 (0.94-1.12) Mod:1.06 (0.99-1.13) Severe: 1.08 (0.96-1.21) |  |  | Cubic age and sex |
|  |  |  |  | Same as above | Linear regression | Adjusted: RR=1.32 (1.26-1.38), mild: 1.18 (1.08-1.29) p=<0.0001, mod: 1.31 (1.23-1.39) p=<0.0001, severe: 1.57 (1.43-1.74) p=<0.0001 | Adjusted: MI RR=0.98 (0.91-1.06) p=0.657 mild: 0.95 (0.82-1.09) Moderate AD: 0.95 (0.86-1.06) Severe: 1.12 (0.94-1.33) |  | Adjusted: RR=1.05 (1.00-1.11) p=0.032 mild: 1.03 (0.94-1.12) Mod:1.05 (0.99-1.13) Severe: 1.08 (0.96-1.21) |  |  | Cubic age, sex, SES of region, access to healthcare |
|  | Cohort | 1,214,133 (35.5% of exposed, 43.43% of unexposed) | 10 | Angina: 1713 (5.33%)  MI: 949 (2.83%)  Non-fatal stroke: 1865 (5.65%) | Linear regression | Age and sex adjusted: RR=1.18 (1.13-1.23) p= <0.0001 mild AD:0.94 (0.85-1.04) p=0.216 Mod:1.21 (1.13-1.29) p=<0.0001 Severe: 1.38 (1.26-1.50) p=<0.0001 | Age and sex adjusted: RR=1.05 (0.98-1.12) p=0.156 mild AD: 0.91 (0.79-1.04) p=0.171 Moderate AD: 1.1 (1.01-1.20) p=0.024 Severe AD: 1.07 (0.94-1.22) p=0.282 |  | Age and sex adjusted: RR= 1.03 (0.98-1.07) p= 0.238 mild: 0.92 (0.84-1.01) p=0.078 Mod: 1.06 (1.00-1.13) p=0.055 Severe: 1.07 (0.98-1.17) p=0.136 |  |  | Cubic age, sex |
|  |  |  |  | Same as above | Linear regression | Adjusted: RR=1.17 (1.12-1.23) p= <0.0001 mild AD:0.93 (0.84-1.03) p=0.19 Mod:1.20 (1.12-1.28) p=<0.0001 Severe: 1.37 (1.25-1.49) p=<0.0001 | Adjusted: RR=1.05 (0.99-1.12) p=0.127 mild AD: 0.92 (0.8-1.05) p= 0.194 Moderate AD: 1.11 (1.02-1.21) p=0.021 Severe AD: 1.08 (0.95-1.22) p=0.255 |  | Adjusted: RR= 1.02 (0.98-1.07) p= 0.346 mild: 0.92 (0.84-1.01) p=0.070 Mod: 1.05 (0.99-1.12) p=0.094 Severe: 1.06 (0.98-1.16) p=0.16 |  |  | Cubic age, sex, SES of region, access to healthcare |
| **Su, 2014^12^** | Cohort | 40,646 (38.1%) | 1-5 |  | Cox proportional hazards |  | Adjusted: HR= 1.31 (95% CI 0.88-1.95) p=0.180 | Adjusted: HR= 1.46 (95% CI 1.10-1.93) p=0.009 | Ischaemic stroke. Crude: HR= 1.29 (95% CI 1.09-1.54) p=0.003 |  |  | Age, sex, partial comorbidities, diagnosis index date |
|  |  |  | 1-5 |  | Cox proportional hazards |  |  |  | Adjusted: HR=1.33 (95% CI 1.12-1.50) p=0.001 |  |  | Age, sex, comorbidities (diabetes, a-fib, HTN, CAD, valvular heart disease, peripheral artery occlusive disease, chronic kidney disease, dyslipidaemia, medications |
| **Sung, 2017^13^** | Cohort | 75,515 (45.8%) | 4-11 |  | Cox proportional hazards regression, Pearson's Chi square test, t-test |  |  |  | Unadjusted: 1.25 (95% CI 1.13-1.39) p=<0.01 |  | Ischaemic stroke. Unadjusted: 1.30 (95% CI 1.17-1.46) p=<0.01 | Age, sex |
|  |  |  | 4-11 |  | Same as above |  |  |  | Adjusted: 1.17 (95% CI 1.06-1.30) |  | Adjusted: 1.21 (95% CI 1.08-1.36) | Age, sex, comorbidity (HTN, diabetes, CAD, a-fib, dyslipidaemia) |
| **Treudler, 2017^14^** | Cross-sectional | 9,481 (43% of exposed, 48.1% of unexposed) |  |  | Mann–Whitney, chi-square or Fisher’s exact test, multiple logistic regression |  | Adjusted: OR 0.5 (95% 0.2-1.2) p=0.268 |  | Adjusted: OR 1.3 (95% 0.6-2.9) p=0.469 |  |  |  |
| **Tsai, 2016^15^** | Cohort | 470,440 (41.87%) | 12 |  | Cox proportional hazards model, log rank test |  |  |  | Crude HR= 1.27 (95% CI=1.23-1.3) p=<0001 |  | Ischaemic stroke. Crude HR=1.3 (95%CI 1.27-1.34) p= <0.0001 | Age, sex comorbidities, medications, treatment |
|  |  |  | 12 |  | Cox proportional hazards model, log rank test |  |  |  | Adjusted HR= 1.17 (95% CI 1.06-1.3) p=<0.0001 |  | Adjusted HR=1.16 (95% CI 1.12-1.19) p=<0.0001 | Same as above |
| **Varbo, 2017^16^** | Cohort | 84,601 | 12 (median: 5) |  | Cox proportional hazards regression |  |  |  | Ischaemic stroke. Age and sex adjusted: HR= 1.24 (95% CI 1.01-1.52, p= 0.04) |  |  | Age, sex |
|  |  |  | 12 (median: 5) |  | Cox proportional hazards regression |  |  |  | Adjusted: HR= 1.19 (95% CI 0.96-1.48, p=0.11) |  |  | Age, sex, hx smoking, pack-years smoking, lipid lowering therapy, LDL cholesterol, HDL cholesterol, triglycerides, diabetes, alcohol, sBP and dBP, BMI, physical activity in leisure/work, a-fib |

*Table Legend:* IRR- incident rate ratios, OR- odds ratio, HR- hazard ratio, RR- risk ratio, SES- socioeconomic status, FmHx- family history, MI- myocardial infarction, HF- heart failure, hx- history, HTN- hypertension, BMI- body mass index, a-fib- atrial fibrillation, IHD- ischaemic heart disease, TIA- transient ischaemic attack, CAD- coronary artery disease, IMD- index of multiple deprivation, sBP- systolic blood pressure, dBP- diastolic blood pressure.

1. **Supplementary table S3. Study definitions of atopic eczema severity**

| First author, year | Definition of Atopic Eczema | | |
| --- | --- | --- | --- |
|  | **MILD** | **MODERATE** | **SEVERE** |
| Andersen, 2016^1^ | Subject did not receive systemic treatment during the study period | N/A | Subject received systemic treatment (azathioprine, methotrexate, cyclosporine, mycophenolate mofetil (MMF)) during the study period |
| Egeberg, 2016^4^* | Subject did not receive systemic treatment | N/A | Subject received systemic treatment (methotrexate, azathioprine, MMF, systemic steroids, PUVA photochemotherapy, cyclosporine) |
| Riis, 2016^8^ | Subject did not receive a prescription for systemic treatment | N/A | Subject received prescription for systemic treatment (cyclosporine, azathioprine or methotrexate) |
| Silverwood and Forbes, 2018^10^ | Assigned as mild disease by default if not fulfilling definition for moderate or severe | i) a second potent topical corticosteroid treatment within one year or ii) a first calcineurin inhibitor treatment | i) a systemic immunosuppressant treatment or ii) a phototherapy code in CPRD or HES, or iii) a referral for atopic eczema |
| Standl, 2016 ^11^** | No anti-inflammatory treatment prescribed | Topical treatment only prescribed (steroids, tacrolimus or pimecrolimus) | Prescribed both topical and systemic treatment (ciclosporin or corticosteroids) |
| Su, 2014^12^ | Defined daily dose (DDD)*** of oral antihistmaines and corticosteroids <28 | N/A | DDD of 28 or more |
| Sung, 2017^13^**** | Number of clinical visits for atopic dermatitis is <10 | Number of clinical visits for atopic dermatitis is 10-19 | Number of clinical visits for atopic dermatitis is 20 or more |

* Not included in Bayesian meta-analysis of atopic eczema severity as cross-sectional in design

**Standl et al report a cross-sectional study (excluded from meta-analysis of atopic eczema severity) and a longitudinal cohort study in single article

***Defined daily dose (DDD) is considered to be the average daily adult maintenance dose of a drug recommended for a given indication. Number of DDDs= total amount of drug / amount of drug in a DDD

****Not included in Bayesian meta-analysis of atopic eczema severity as their definition of severity groupings were fundamentally different from the other studies', making the groups unpoolable

1. **Risk of bias in individual studies using the Newcastle Ottawa Scale (NOS)**

Overall, the majority of included studies in the present systematic review were found to have low to moderate risk of bias from the Newcastle-Ottawa Scale. In the confounding assessment, 11 studies were found to have low risk, 3 had unclear risk, and 2 studies were deemed high risk due to lack of adjustment. With respect to participant selection, 13 studies were found to have low risk, 2 studies had moderate risk due to selectivity of participant cohorts (exclusively female registered nurses, or those that had to respond to an invitation), and one study was found to be high risk (where only 33% of invited patients participated in the study). In the assessment of misclassification of variables, 9 studies were found to be low risk, 2 studies with moderate risk (use of structured medical record and routine care, or reliant on self-reported data and routine medical care), and 5 studies were of high risk of bias (study data from non-validated self-reported records and possible recall bias, or non-validated insurance claims data). With respect to bias due to missing data, 4 studies were determined to be low risk, 5 with unclear risk, 2 with moderate risk (cross-sectional studies with moderate exclusion of patients with missing data), and 2 with high risk of bias (case-control study with no inclusion of patients with missing data, cross-sectional design with no analysis of impact of missing data and low responder rate).

**Supplementary table S4. Detailed risk of bias based on the Newcastle Ottawa Scale (NOS)**

|  | **Confounding** | **Selection of participants** | | **Misclassification of variables** | | | | | | | | | | **Bias due to missing data** | | **Reverse causation** | **Generalisability** |
| --- | --- | --- | --- | --- | --- | --- | --- | --- | --- | --- | --- | --- | --- | --- | --- | --- | --- |
| **First author, publication year** | **Age and other confounders** | **Participation bias** | **Selection of controls** | **Exposure** | | | | **Outcome** | | | | **Covariates** | |  |  |  |  |
|  |  |  |  | **Differential** | | |  | **Differential** | | |  |  |  |  |  |  |  |
|  |  |  |  | **Recall bias** | **Observer bias** | **Ascertainment bias** | **Non-differential** | **Recall bias** | **Observer bias** | **Ascertainment bias** | **Non-differential** | **Differential** | **Non-differential** | **Differential loss to follow up** | **Exclusion of individuals with missing data** |  |  |
| **Andersen, 2016^1^** | Low: adjusted for age, sex, and other confounders | Low: automated participation | Low: selected at random from same population as cases | Low: structured record | Low: exposure status defined by observer blinded to outcome status | Low: method of data collection unlikely to affect results | High: relies on routine medical care | Low: structured record | Low: outcome status defined after exposure status assigned | Low: ascertainment of outcome unlikely to be influenced by exposure status | Low: use of ICD codes to validate diagnosis MI and ischemic stroke had been validated | Moderate: smoking, alcohol consumption and BMI data likely to have errors in this registry | Moderate: administrative errors in routinely collected data | Low: automated follow up | Low: 385 exposed subjects excluded due to lack of controls | Low: cohort study | B: hospital diagnosed atopic eczema only, excluding those managed in community |
| **Drucker, 2016^2^** | Low: adjusted for age (per 5 years) and other confounders | High: cohort of exclusively female registered nurses, responding to survey | Low: selected at random from same population as cases | High: self-reported | Moderate: self-reported exposure status | Low: method of data collection unlikely to affect results | High: relies on routine medical care therefore may be missed | High: self-reported | Low: Outcome self-reported and confirmed by medical record review | Low: ascertainment of outcome unlikely to be influenced by exposure status | Moderate: self-report was confirmed by medical records | High: self-reported, likely underestimate of smoking, BMI, alcohol consumption | Unclear | N/A as cross-sectional design | Moderate: 13% individuals have data missing with no analysis of impact | High: cross-sectional design | C: study population of predominantly white, female nurses from USA |
| **Drucker, 2017^3^** | Low: adjusted for age, sex, and other confounders | High: participants have responded to invitation | Low: selected from same population as cases | High: self-reported | Unclear: self-reported exposure status and Canadian Partnership for Tomorrow Project cohort (unclear if there was observer blinding) | Low: method of data collection unlikely to affect results | High: relies on routine medical care | High: self-reported | Moderate: Self-reported outcome | Low: ascertainment of outcome unlikely to be influenced by exposure status; authors stated that self-reported outcomes have good agreement with medical records | Moderate: self-reported; validation study was conducted on self-reported diseases in the Canadian Partnership for Tomorrow project cohort but not reported | High: self-reported on covariates | Unclear | N/A as cross-sectional design | Moderate: 15% excluded with no analysis of impact | Low: participant included if diagnosis of atopic eczema preceded cardiovascular  outcomes | B: volunteer based participation (may be more health conscious cohort), definition of cases could include types of eczema other than atopic eczema |
| **Egeberg, 2016^4^** | Low: adjusted for age, sex, and other confounders | Low: automated participation | Low: selected at random from same population as cases | Low: structured record | Low: exposure status defined by observer blinded to outcome status | Low: method of data collection unlikely to affect results | High: relies on routine medical care | Low: structured record | Low: Outcome status defined by blinded observer to exposure status | Low: ascertainment of outcome unlikely to be influenced by exposure status | Moderate: non-validated method; diagnostic codes from registries used | Low: covariates chosen unlikely to be subject to differential error | Moderate: administrative errors in routinely collected data | N/A as cross-sectional design | Unclear: no mention of missing data | High: cross-sectional design | B: hospital diagnosed atopic eczema only, excluding those managed in community |
| **Kwa, 2017^5^** | Low: adjusted for age, sex, and other confounders | Low: automated participation | Low: selected at random from same population as cases | Low: structured record | Low: exposure status defined by observer blinded to outcome status | Low: method of data collection unlikely to affect results | High: relies on routine medical care | Low: structured record | Low: Outcome status defined by observer blinded to exposure status | Low: ascertainment of outcome unlikely to be influenced by exposure status | High: authors state outcome status likely to be improperly captured in this dataset | Unclear | Moderate: administrative errors in routinely collected data | N/A as cross-sectional design | Unclear: no mention of missing data | High: cross-sectional design | C: Inpatient hospitalisations only |
| **Marshall, 2013^6^** | Unclear: no detail on how age was adjusted for | Low: automated participation | Low: selected at random from same population as cases | High: insurance claims data | Low: exposure status defined by observer blinded to outcome status | Low: method of data collection unlikely to affect results | High: relies on routine medical care | Low: structured record | Low: Outcome status defined by observer blinded to exposure status | Low: ascertainment of outcome unlikely to be influenced by exposure status | Moderate: non-validated method; diagnostic codes from registries used | Low: limited covariates chosen | Unclear | Unclear: described as case-control study however designed as very restricted cohort study | High: Case-control study; did not included patients with missing data | High: 28% of possible ‘cases’ has missing medication records | B/C: restrictive criteria of >30 year olds with new diagnosis of atopic eczema requiring ‘one year of no disease related treatment no disease related treatment prior to their diagnosis’ |
| **Radtke, 2017^7^** | High: no adjustment | Low: automated participation | Low: selected from same population as cases | Low: structured record | Low: exposure status defined by observer blinded to outcome status | Low: method of data collection unlikely to affect results | High: relies on routine medical care | Low: structured record | Low: Outcome status defined by observer blinded to exposure status | Low: ascertainment of outcome unlikely to be influenced by exposure status | Unclear: no detail on method of determining outcome or whether it is validated | N/A no covariates | N/A no covariates | N/A as cross-sectional design | Unclear: no mention of missing data | High: cross-sectional design | A: requires hospital, ambulatory or disability related diagnoses |
| **Riis, 2016^8^** | Low: adjusted for birth year in three categories of 10 years | Low: automated participation | Low: selected from same population as cases | Low: structured record | Low: exposure status defined by observer blinded to outcome status | Low: method of data collection unlikely to affect results | High: relies on routine medical care | Low: structured record | Low: outcome status defined after exposure status assigned | Low: ascertainment of outcome unlikely to be influenced by exposure status | Moderate; non-validated method; diagnostic codes from registries used | Low: covariates chosen unlikely to be subject to differential error | Moderate: administrative errors in routinely collected data | Low: automated follow up | Low: authors state almost complete information available | Low: cohort study | B: hospital diagnosed atopic eczema only, excluding those managed in community |
| **Silverberg, 2015^9^** | Unclear: states adjusted for age but with no detail | Low: surveys randomised and weighted to be representative | Low: selected from same population as cases | High: self-reported | Low: trained interviewers, survey not specifically designed to answer this question | Low: method of data collection unlikely to affect results | High: NHIS ’12 relies on routine medical care otherwise self-reported | High: self-reported | Low: trained interviewers, survey not specifically designed to answer this question | Low: ascertainment of outcome unlikely to be influenced by exposure status | High: non-validated method; self-reported | High: self-reported, likely underestimate of smoking, BMI, alcohol consumption, and overestimate exercise | Low: Comprehensive survey data with trained interviewers and computer-assisted personal interviewing | N/A as cross-sectional design | Low: small amount of data missing | High: cross-sectional design | B: definition of cases could include types of eczema other than atopic eczema |
| **Silverwood and Forbes, 2018^10^** | Low: adjusted for age, sex, and other confounders | Low: automated participation | Low: selected from same population as cases | Low: structured record | Low: exposure status defined by observer blinded to outcome status | Low: method of data collection unlikely to affect results | Low: validated with 82% positive predicted value | Low: structured record | Low: outcome status defined after exposure status assigned | Low: ascertainment of outcome unlikely to be influenced by exposure status | Low: validated method | Low: covariates likely to be well estimated in this dataset | Moderate: administrative errors in routinely collected data | Low: automated follow up | Low: 27.3% excluded but sensitivity analysis conducted | Low: cohort study | A: includes primary and secondary care and one diagnosis (with two treatments) required for definition |
| **Standl, 2016^11^** | Low: adjusted for age, sex, and other confounders | Low: automated participation | Low: selected from same population as cases | Low: structured record | Low: exposure status defined by observer blinded to outcome status | Low: method of data collection unlikely to affect results | High: relies on routine medical care | Low: structured record | Low: outcome status defined after exposure status assigned | Low: ascertainment of outcome unlikely to be influenced by exposure status | Moderate: non-validated method; diagnostic codes from registries used | Moderate: general socioeconomic characteristics and healthcare access inferred from post code | Moderate: administrative errors in routinely collected data | Low: automated follow up | Unclear: no mention of missing data | Low: cohort study (cross-sectional analysis also reported which would be at high risk) | B: No data from primary care, excluding those managed in community |
| **Su, 2014^12^** | Low: adjusted for age, sex, and other confounders | Low: automated participation | Low: selected from same population as cases | Low: structured record | Low: exposure status defined by observer blinded to outcome status | Low: method of data collection unlikely to affect results | High: relies on routine medical care | Low: structured record | Low: outcome status defined after exposure status assigned | Low: ascertainment of outcome unlikely to be influenced by exposure status | Moderate: non-validated method; diagnostic codes from registries used | Low: covariates chosen unlikely to be subject to differential error | Moderate: administrative errors in routinely collected data | Low: automated follow up | Unclear: no mention of missing data | Low: cohort study | A: data from primary and secondary care included, although required new diagnoses aged >20 |
| **Sung, 2017^13^** | Low: adjusted for age, sex, and other confounders | Low: automated participation | Low: selected from same population as cases | Low: structured record | Low: exposure status defined by observer blinded to outcome status | Low: method of data collection unlikely to affect results | High: relies on routine medical care | Low: structured record | Low: outcome status defined after exposure status assigned | Low: ascertainment of outcome unlikely to be influenced by exposure status | Moderate: non-validated method; diagnostic codes from registries used | Low: covariates chosen unlikely to be subject to differential error | Moderate: administrative errors in routinely collected data | Low: automated follow up | Unclear: no mention of missing data | Low: cohort study | B: data from primary and secondary care included, required three consensus diagnoses which may exclude milder cases |
| **Treudler, 2017^14^** | High: no adjustment | High: 33% participation of those invited | Unclear | High: self-reported | Low: structured interview, survey not specifically designed to answer this question | Low: method of data collection unlikely to affect results | High: relies on routine medical care (self-report of physician diagnosis) | High: self-reported | Unclear | Unclear | High: non-validated method; self-reported | High: self-reported, likely underestimate of smoking | Unclear | N/A as cross-sectional design | High: significant amount of missing data with no analysis of impact | High: cross-sectional design | C: low responder rate to study, responders likely to be a selective group e.g. health conscious |
| **Tsai, 2016^15^** | Unclear: states adjusted for age but with no detail | Low: automated participation | Low: selected from same population as cases | Low: structured record | Low: exposure status defined by observer blinded to outcome status | Low: method of data collection unlikely to affect results | High: relies on routine medical care | Low: structured record | Low: outcome status defined after exposure status assigned | Low: ascertainment of outcome unlikely to be influenced by exposure status | Moderate: non-validated method; diagnostic codes from registries used | Low: covariates chosen unlikely to be subject to differential error | Moderate: administrative errors in routinely collected data | Low: automated follow up | Unclear: no mention of missing data | Low: cohort study | B: definition of exposure includes types other than atopic eczema |
| **Varbo, 2017^16^** | Low: adjusted for age, sex, and other confounders | Low: large community based cohort study | Low: selected from same population as cases | High: self-reported | Low: trained interviewers, survey not specifically designed to answer this question | Low: method of data collection unlikely to affect results | Moderate: Relies on a combination of routine medical care and prospective study data | Low: medical records reviewed by two blinded physicians | Low: outcome status defined after exposure status assigned | Low: ascertainment of outcome unlikely to be influenced by exposure status | Moderate: non-validated method; diagnostic codes from registries used | High: self-reported on covariates | Unclear | Low: automated follow up | Unclear: no mention of missing data | Low: cohort study | B: self-reported definition of exposure could encompass contact dermatitis |

*Table legend:* Assessment of generalisability: A) truly representative of the average subject in the community B) somewhat representative of the average subject in the community, C) selected group of users e.g. nurses, volunteers, D) no description of the derivation of the cohort. MI= myocardial infarction

1. **Reasons to up- or down-grade quality of evidence in Grading of Recommendations, Assessment, Development and Evaluation (GRADE)**

1) Risk of bias:

- Not serious if >50% of studies have no domain which is at high risk of bias.

- Serious if studies are judged to be between ‘not serious’ and ‘very serious’.

- Very serious if studies which have two or more domains at high risk of bias represent more than 50% of the total studies and contribute more than 50% to any meta-analyses.

2) Inconsistency:

- Not serious if have 0, serious if have 1, and very serious if have two or more of the following:

- Heterogeneity is moderate (I² ~30-60%), or above.

- Wide variance of point estimates across different studies.

- Minimal overlap of confidence intervals.

3) Indirectness:

Not serious if have 0, serious if have 1, and very serious if have two of the following:

- An indirect comparison (for example study A compares to a placebo and study B compares to a different drug).

- Studies differ in terms of population (e.g. hospital inpatients only vs population based survey)

- Studies differ in terms of exposure definition (e.g. for example use different methods to ascertain atopic eczema exposures).

- Studies differ in terms of outcome measures (e.g. for example be restricted to certain time-frames or have a different definition of a cardiovascular outcome).

4) Imprecision:

- Serious imprecision: Wide confidence intervals

- Very serious imprecision: Very wide confidence intervals

Upgrading reasons include

5) Large effect:

- None: most effect estimates <2

- Strong association: effect estimates >2 or <0.5 (based on direct evidence, with no plausible confounders)

- Very strong association: effect estimates >4 or <0.2 (based on direct evidence with no serious problems with risk of bias or precision, i.e. with (sufficiently narrow confidence intervals).

6) Plausible confounding

- Would dilute the demonstrated effect: e.g. If, for instance, only sicker patients receive an intervention or exposure, yet they still fare better, it is likely that the actual intervention or exposure effect is even larger than the data suggest (confounding by indication).

- Would suggest spurious effect: When confounding is expected to increase the effect but no effect was observed.

7) Dose response gradient

- Yes

- No

1. **GRADE assessments and explanation**

Table S4 provides our GRADE assessment by each cardiovascular outcome. In GRADE, non-randomised studies provide low quality evidence for outcomes at the beginning of the quality assessment. Our confidence in the evidence for an outcome can then be up- or down-graded according to different aspects of evidence quality. See the supplementary information for further detail on up- or down-grading. Stroke, MI, and heart failure demonstrated inconsistency (or high heterogeneity). Angina and ischaemic stroke appeared to be consistent however, three of the four cohort studies looking at ischaemic stroke used the same Taiwanese dataset. It is important to note that heterogeneity is poorly estimated where there are few studies.

Indirectness (the variability of the populations, exposures and outcomes between study) was very serious for stroke, ischaemic stroke, angina, MI, and heart failure because definitions of atopic eczema and of cardiovascular disease (CVD) were varied, with some being self-reported and some being based on clinical codes in administrative data (for detail see Table S1). Self-reported definitions of atopic eczema may have inadvertently included other types of eczema such as contact dermatitis. Populations differed as some routinely collected data sets excluded patients in primary care, and other studies were population based. Imprecision (wide confidence intervals) was significant for all outcomes apart from arrhythmias, coronary revascularisation and cardiovascular death. Angina, MI and heart failure were upgraded for demonstrating a significant dose-response, whilst coronary revascularisation, arrhythmias and cardiovascular death were not upgraded (despite a significant dose response found for cardiovascular death), as these outcomes were only examined in one or two studies.

**Supplementary table S5. GRADE quality assessment**

| **Quality assessment** | | | | | | | |
| --- | --- | --- | --- | --- | --- | --- | --- |
| **Outcome** | **Downgrade** | | | | | **Upgrade** | **Quality** |
|  | **Design and number of studies** | **Risk of bias** | **Inconsistency** | **Indirectness** | **Imprecision** |  |  |
| Angina | 2 cohort, 4 cross-sectional | Not serious | Not serious | Very serious indirectness (because of indirectness of outcome and exposure) | Very serious imprecision | Upgrade: Dose response relationship present | ⊕⊕⊕⊕  Very low |
| MI | 5 cohort, 9 cross-sectional | Not serious | Very serious | Very serious indirectness (because of indirectness of populations, outcome and exposure) | Very serious imprecision | Upgrade: Dose response relationship present | ⊕⊕⊕⊕  Very low |
| Coronary revascularisation | 1 cohort | Not serious | * | * | No serious imprecision | * | **⊕⊕**⊕⊕  Low |
| Heart failure | 2 cohort, 2 cross-sectional | Not serious | Very serious | Very serious indirectness (because of indirectness of populations, outcome and exposure) | Very serious imprecision | Upgrade: Dose response relationship present | ⊕⊕⊕⊕  Very low |
| Arrhythmias | 1 cohort | Not serious | * | * | No serious imprecision | * | **⊕⊕**⊕⊕  Low |
| Stroke | 4 cohort, 9 cross-sectional | Not serious | Very serious | Very serious indirectness (because of indirectness of populations, outcome and exposure) | Very serious imprecision |  | ⊕⊕⊕⊕  Very low |
| Ischaemic Stroke | 5 cohort | Not serious | Not serious | Very serious indirectness (because of indirectness of outcome and exposure) | Serious imprecision |  | ⊕⊕⊕⊕  Very low |
| Cardiovascular death | 2 cohort | Not serious | * | * | No serious imprecision | * | **⊕⊕**⊕⊕  Low |

* Could not be assessed for small number of studies

1. **Supplementary table S6. Detail of models of adjustment for studies combined in the meta-analyses**

| First author, year | No adjustment | Minimally adjusted | Fully adjusted (confounders) | Mixed mediation/confounder model |
| --- | --- | --- | --- | --- |
| Andersen, 2016^1^ |  | ✔ age and sex |  | ✔ age, sex, SES, smoking, medication, comorbidities |
| Drucker, 2016^2^ |  | ✔ age |  | *✔ Age, ethnicity, BMI, physical activity, alcohol, smoking, FH of MI, hormone replacement use  ✔ Age, ethnicity, BMI, physical activity, alcohol, smoking, FH of MI, hormone replacement use, hx of hypertension, high cholesterol, diabetes |
| Drucker, 2017^3^ |  | ✔ age and sex |  | *✔ age, sex, ethnic background, BMI, history of ever smoking 100 cigarettes, weekly alcohol intake, average daily sleep, physical activity, asthma  ✔ age, sex, ethnic background, BMI, history of ever smoking 100 cigarettes, weekly alcohol intake, average daily sleep, physical activity, asthma, AND additionally adjusted for each of the other three outcomes (e.g. for the analysis of hypertension, additionally adjusted for T2D, MI and stroke) |
| Egeberg, 2016^4^ |  |  | ✔ age, sex, socioeconomic status, number of dermatology visits |  |
| Kwa, 2017^5^ | ✔ |  | ✔ age,sex, race/ethnicity, mean annual household income, insurance status, number of chronic conditions, and hospital region |  |
| Riis, 2016^8^ | ✔ (matched on sex, birth year) |  |  | ✔ gender, birth-year category, educational level, diabetes mellitus, hypertension, hyperlipidaemia or stroke |
| Silverberg, 2015 (NHANES)^9^ |  |  | ✔ age, highest level of edu- cation in the household, household income, race/ethnicity, and sex **(model i)** | ✔ BMI, history of ever smoking cigarettes, consumption of alcohol in the past year, and vigorous activity in the past 30 days **(model iii)** |
| Silverberg, 2015 (NHIS 2010)^9^ |  |  | ✔ age, highest level of edu- cation in the household, household income, race/ethnicity, and sex **(model i)** | ✔ BMI, history of ever smoking cigarettes, consumption of alcohol in the past year, and vigorous activity in the past 30 days **(model iii)** |
| Silverberg, 2015 (NHIS 2012)^9^ |  |  | ✔ age, highest level of edu- cation in the household, household income, race/ethnicity, and sex **(model i)** | ✔ BMI, history of ever smoking cigarettes, consumption of alcohol in the past year, and vigorous activity in the past 30 days **(model iii)** |
| Silverwood and Forbes, 2018^10^ | ✔ (matched on age at cohort entry, gender, date at cohort entry and practice) |  | ✔ current calendar period (1997-1999, 2000-2004, 2005-2009, 2010-2015), time since diagnosis (0-4, 5-9, 10-14, 15-19, 20+ years), IMD at cohort entry, and time-varying asthma | ✔ current calendar period (1997-1999, 2000-2004, 2005-2009, 2010-2015), time since diagnosis (0-4, 5-9, 10-14, 15-19, 20+ years), IMD at cohort entry, and time-varying asthma, BMI and smoking at cohort entry, time-varying hyperlipidaemia, hypertension, depression, anxiety, diabetes and severe alcohol use |
| Standl, 2016 (Cross-Sectional)^11^ |  | ✔ Cubic age and sex | ✔ sex, cubic age, and socioeconomic status of region and access to health care |  |
| Standl, 2016 (Cohort)^11^ |  | ✔ Cubic age and sex | ✔ sex, cubic age, and socioeconomic status of region and access to health care |  |
| Su, 2014^12^ | ✔ (matched on age, sex, partial comorbidities, diagnosis index date) |  |  | ✔ age and sex and comorbidities (diabetes, AF, HTN, coronary artery disease, valvular heart disease, peripheral artery occlusive disease, chronic kidney disease, dyslipidaemia), medications (warfarin, Statins, ACEI/ARBs, Aspirin/clopidogrel) |
| Sung, 2017^13^ | ✔ (matched on age and sex) |  |  | ✔ Age, sex, comorbidity (HTN, DM, CAD, AF, and hyperlipidemia) |
| Treudler, 2017^14^ |  |  |  | ✔ age, sex, BMI, smoking, SES, autoimmune disease, rheumatic disease, coronary artery disease, MI, stroke, IBD |
| Tsai, 2016^15^ | ✔ (pair matched on age, sex, index year) |  |  | ✔ age, sex, treatment (systemic corticosteroids, traditional chinese medicine) and comorbidities (DM, HT, hyperlipidaemia, AF) |
| Varbo, 2017^16^ |  | ✔ age and sex |  | ✔ age, sex, current and former smoking, pack years, lipid lowering therapy, LDL cholesterol, HDL cholesterol, triglycerides, diabetes, alcohol consumption, systolic and diastolic BP, BMI, physical activity in leisure and work, atrial fibrillation |

*The estimate marked with an asterisk was chosen for the meta-analysis where more than one model of adjustment was presented

1. **Detail on statistical methods**

**Random-effects models:**

Random-effects models assume the log odds ratios (ORs) estimate effects in multiple populations that follow a normal distribution with a mean and standard deviation rather than estimating one fixed population value. This model is usually appropriate for meta-analysing observational studies where multiple populations could be expected due to, for example, differences in defining atopic eczema, or differences in adjustment strategies.

**Statistical methods for the association between atopic eczema and the risk of CVD outcomes:**

For cross-sectional studies we displayed the extracted odds ratios of unstable angina, heart failure, myocardial infarction and stroke (separately) for participants with atopic eczema compared to participants without atopic eczema in forest plots. We used a random effects meta-analysis model with the inverse variance method of DerSimonian and Laird^17^ to estimate between-study (population) heterogeneity and the I-squared measure to quantify the proportion of total variance attributable to this heterogeneity^18^. The mean can be thought of as a “typical” (log) OR, while the standard deviation quantifies their underlying between-population variability. We present the 95% confidence interval (CI) for the precision of the mean, which disregards the between-population variability, as well as a 95% prediction interval (PI). The PI additionally accounts for this and is the range in which 95% of underlying population-specific ORs are predicted to lie^19^. As the CI can convey falsely precise confidence in the presence of population heterogeneity, we primarily interpret the PIs to address our research question.

We performed a similar analysis in cohort studies, pooling the log hazard ratios and/or log incident rate ratios in the meta-analysis for the same outcomes with additional assessment of the relationship between atopic eczema and cardiovascular death and ischaemic stroke.

## **Statistical methods for the association between atopic eczema and the risk of CVD outcomes by atopic eczema severity:**

All the studies assessing the relationship between atopic eczema severity and cardiovascular outcomes were cohorts, with the exception of two cross-sectional studies. We excluded these (Egeberg et al^4^, cross-sectional study reported in Standl et al^11^, and Sung et al’s cohort study^13^, as their severity groupings were fundamentally different from the other studies', making the groups unpoolable) displaying the rest of the studies in a graph of the (log) hazard ratio (HR) or incidence rate ratio (IRR) against atopic eczema severity (mild, moderate or severe). Because it is on the log scale, this shows the accumulation of excess CVD risk in people who have atopic eczema from the baseline level of risk in people without atopic eczema. Two studies analysed the association between myocardial infarction and atopic eczema by severity using the same dataset with the same study period (Andersen et al^1^ and Riis et al^8^). We included the study with a stricter definition of atopic eczema exposure and slightly longer follow up time^8^, and conducted a sensitivity analysis to explore any potential impact of this choice.

To estimate the relative increase in risk attributable to severity, we constructed a multivariate linear mixed model relating the log HRs (or IRRs) for each of the six outcomes to atopic eczema severity. The linear predictor consisted of an outcome-specific fixed intercept, a random study intercept, an outcome-specific fixed slope for severity (assuming linearity) and random error. We performed a Bayesian analysis on this model with uninformative ‘priors’ (assumptions about the value of unknown parameters that affect estimation), except for the precision of the observed log HRs (IRRs), which we gave the informative priors of the inverse of their observed variance. We used a Bayesian model because of the relative ease of both incorporating this uncertainty around the outcomes within a linear mixed model and jointly modelling multiple outcomes. We report the means and 2.5 and 97.5 quantiles of the posterior distributions, which are the pooled estimates and their 95% credibility intervals (CrI, analogous to the 95% confidence interval for the mean). We used a Bayesian instead of a frequentist method because correct inference can be achieved with smaller samples than are necessary for frequentist mixed-effects models^20^.

We performed a secondary analysis to estimate the generalised increase in risk attributable to atopic eczema severity. Here we assumed that the risk increases in the outcomes we studied adequately represents the distribution of this effect across all cardiovascular outcomes. Using a similar Bayesian model, we changed the fixed outcome intercept to a random intercept, and the fixed outcome slopes to a single fixed slope with a random slope effect. The variance of the random slope effects estimates the between-outcome variability in risk increase, and can be used to estimate the distribution of risk increase across all CV outcomes.

We report the means and 2.5 and 97.5 quantiles of the atopic eczema severity slope distributions. The latter two statistics form a 95% credibility interval (CrI), which is analogous to the 95% confidence interval for the mean. For the second model we additionally estimate the range of risk increases that would be observed across all cardiovascular outcomes by summing the slope and its estimated random effect, reporting the 2.5 and 97.5 quantiles of this posterior distribution. This is the 95% uncertainty interval, analogous to the 95% prediction interval described above. The code for both models is available in the appendix. No subgroup analyses were undertaken.

Both models used the JAGS (Just Another Gibbs Sampler) algorithm, allowing the sampler a 1000-run adaptation period during 5000 burn-in iterations followed by 100,000 MCMC (Markov-Chain Monte Carlo) iterations thinned to every 5^th^, resulting in posterior distributions of 20,000 samples from which to draw inference.

All statistical analyses were performed in R v3.4.3^21^ using the “meta”^22^ and “rjags”^23^ packages.

1. **Detail on statistical results**

**The association between atopic eczema and the risk of CVD outcomes:**

Forest plots for cross-sectional studies are in Figure 1 in the main manuscript. For angina, the pooled OR between three studies’ maximally adjusted estimates was 1.78, 95% CI (1.44 to 2.20). Although the I^2 was estimated to be zero, the 95% prediction interval (PI) (0.45 to 7.03) was much wider. This is because the between-study heterogeneity was estimated so the prediction interval is constructed using quantiles from the t-distribution rather than the normal distribution, and the t-value on two degrees of freedom is much larger than the corresponding z-value (4.30 vs. 1.96). For heart failure, the pooled OR between two studies was 1.32, 95% CI (0.70 to 2.48). I^2 was 74% and the 95% PI was not estimated due to too few studies.

Eight studies provided estimates for the association between atopic eczema and each of MI and stroke. For both outcomes there was substantial heterogeneity between studies (I squared 97 and 96%, respectively), and there was no evidence that the pooled OR was different from 1 (MI: 1.04, 95% CI (0.71 to 1.54); stroke: 1.18, 95% CI (0.83 to 1.66)). Both prediction intervals were wide (0.27 to 4.10 and 0.35 to 3.89 respectively), suggesting a wide range of population effects, some of which may associate having atopic eczema with lower odds and some with higher odds of having an MI or stroke.

Forest plots for cohort studies are in Figure 2 in the main manuscript. Few studies provided estimates for the effect of atopic eczema on the risk of angina, heart failure and cardiovascular death. The pooled relative risk for angina was estimated to be 1.18, 95% CI (1.13 to 1.24), and for heart failure was 1.26, 95% CI (1.05 to 1.51). Both were estimated from two studies. I^2 was 2 and 46% respectively but prediction intervals were not estimated due to the small number of studies. The only study to report an effect of atopic eczema on cardiovascular death was Silverwood & Forbes^10^, which provided no evidence of an association (HR 0.98, 95% CI 0.91 to 1.05).

Four studies provided estimates for the effect of atopic eczema and the risk of (each of) ischaemic stroke, MI and stroke. There was evidence that both the pooled effect and the distribution of population effects from which estimates were derived supported an increased risk of ischaemic stroke (pooled RR 1.17, 95% CI 1.14 to 1.20, 95% PI 1.10 to 1.24). For MI and stroke, there was evidence that the pooled association was an increase in risk, but not across the entire population distribution (MI: average RR 1.12, 95% CI 1.00 to 1.25, 95% PI 0.73 to 1.70; stroke average RR 1.10, 95% CI 1.03 to 1.17, 95% PI 0.83 to 1.45).

**The association between atopic eczema severity and the risk of CVD outcomes:**

The risk of developing all six outcomes appeared to increase with increasing atopic eczema severity, and the slope of this relationship appeared very similar between studies and between outcomes (Figure 3A in the main manuscript). All six slopes estimated from the multivariate Bayesian model were positive, indicating an increasing risk of CVD with increasing atopic eczema severity (Figure 3B in the main manuscript). Atopic eczema severity was associated with an increased risk of angina (relative risk (RR) per unit increase in severity 1.16, 95% CrI 1.07 to 1.26), heart failure (RR 1.20, 95% CrI 1.06 to 1.36), cardiovascular death (RR 1.25, 95% CrI 1.13 to 1.38) and MI (RR 1.14, 95% CrI 1.05 to 1.23) in people with atopic eczema. There was no statistically significant evidence for the association with stroke (RR 1.07, 95% CrI 0.99 to 1.15) or ischaemic stroke (RR 1.17, 95% CrI 0.98 to 1.39). These results were not materially altered by including Andersen^1^ rather than Riis^8^ in the analysis, and no conclusions changed (data not shown).

The mean relative risk increase of developing any cardiovascular outcome was 1.15, 95% CrI 1.09 to 1.21. The uncertainty interval where we expect 95% of all RRs to be was 1.04 to 1.28, providing evidence for an association, in people with atopic eczema, between severity of disease and an increased risk of cardiovascular disease.

**Additional analyses of heterogeneity and publication bias:**

We have not explored causes of heterogeneity because the low number of studies for these outcomes provides little power to detect them. We also could not explore publication bias with funnel plots, because in the presence of high heterogeneity there is no reason to expect a plot of estimates against their standard errors to have a funnel shape.

1. **Bayesian model for estimating atopic eczema severity and the risk of six cardiovascular outcomes**

jcode <- "model{

# Likelihood

for(i in 1:N){ #number of studies

for(j in 1:M){ #number of exposures (3x levels of eczema severity)

for(k in 1:P){ #number of outcomes

logor[i,j,k] ~ dnorm(mu[i,j,k], pow(se[i,j,k],-2))

mu[i,j,k] <- b0[i] + b1[k]*exposure[j] + b2[k] + e[i,j,k]

e[i,j,k] ~ dnorm(0,inv.sigma2)

}

}

}

#model for missing SE data (hard coded, JAGS doesn't cope with partially observed independent variables)

for(i in c(1,2,5)){

for(j in 1:3){

for(k in 1:1){

se[i,j,k] <- pow(se2[i,j,k],0.5)

se2[i,j,k] ~ dunif(0, 0.2)

}

}

}

for(i in c(1,2,4,5)){

for(j in 1:3){

for(k in 2:2){

se[i,j,k] <- pow(se2[i,j,k],0.5)

se2[i,j,k] ~ dunif(0, 0.2)

}

}

}

for(i in c(2,4,5)){

for(j in 1:3){

for(k in 3:3){

se[i,j,k] <- pow(se2[i,j,k],0.5)

se2[i,j,k] ~ dunif(0, 0.2)

}

}

}

for(i in c(2:4)){

for(j in 1:3){

for(k in 4:4){

se[i,j,k] <- pow(se2[i,j,k],0.5)

se2[i,j,k] ~ dunif(0, 0.2)

}

}

}

for(i in c(1,5)){

for(j in 1:3){

for(k in 5:5){

se[i,j,k] <- pow(se2[i,j,k],0.5)

se2[i,j,k] ~ dunif(0, 0.2)

}

}

}

for(i in c(1,2,5)){

for(j in 1:3){

for(k in 6:6){

se[i,j,k] <- pow(se2[i,j,k],0.5)

se2[i,j,k] ~ dunif(0, 0.2)

}

}

}

for(i in 1:1){

for(j in 1:1){

for(k in 3:4){

se[i,j,k] <- pow(se2[i,j,k],0.5)

se2[i,j,k] ~ dunif(0, 0.2)

}

}

}

for(i in 2:2){

for(j in 1:1){

for(k in 5:5){

se[i,j,k] <- pow(se2[i,j,k],0.5)

se2[i,j,k] ~ dunif(0, 0.2)

}

}

}

# Random study effect distribution

for(i in 1:N){

b0[i] ~ dnorm(mu.b0,inv.tau2)

}

# Precisions

inv.sigma2 <- 1/sigma2

inv.tau2 <- 1/tau2

# Priors

sigma2 ~ dunif(0, 0.005) # residual variance

tau2 ~ dunif(0, 0.005) # random intercept variance

mu.b0 ~ dnorm(0,0.001) # mean beta0 coefficient

for (k in 1:P) {

b1[k] ~ dnorm(0,0.001) #slope for each outcome

b2[k] ~ dnorm(0,0.001) #intercept for each outcome

}

#PREDICTIONS #############

# Slope of eczema exposure for each outcome

ang <- b1[1]

ccf <- b1[2]

cvd <- b1[3]

iss <- b1[4]

mi <- b1[5]

str <- b1[6]

}"

jdat <- list(N=length(levels(d3$study)), M=length(levels(d3$exposure)), P=length(levels(d3$outcome)),

logor=array(d3$logor, dim = c(length(levels(d3$study)),

length(levels(d3$exposure)),

length(levels(d3$outcome)))),

se=array(d3$se, dim = c(length(levels(d3$study)),

length(levels(d3$exposure)),

length(levels(d3$outcome)))),

exposure=1:length(levels(d3$exposure)))

set.seed(1000399)

jmod <- jags.model(textConnection(jcode), data=jdat, n.chains=2, n.adapt=1000)

update(jmod,5000)

jpos <- coda.samples(jmod, c("ang", "ccf","cvd","iss","mi","str"), n.iter=100000, thin=5)

1. **Bayesian model for estimating atopic eczema severity and the average risk for cardiovascular outcomes**

jcode2 <- "model{

# Likelihood

for(i in 1:N){ #number of studies

for(j in 1:M){ #number of exposures (eczema severity)

for(k in 1:P){ #number of outcomes

logor[i,j,k] ~ dnorm(mu[i,j,k], pow(se[i,j,k],-2))

mu[i,j,k] <- b0[i] + b1[k]*exposure[j] + b2[k] + e[i,j,k]

e[i,j,k] ~ dnorm(0,inv.sigma2)

}

}

}

#model for missing SE data (hard coded, JAGS doesn't cope with partially observed independent variables)

for(i in c(1,2,5)){

for(j in 1:3){

for(k in 1:1){

se[i,j,k] <- pow(se2[i,j,k],0.5)

se2[i,j,k] ~ dunif(0, 0.2)

}

}

}

for(i in c(1,2,4,5)){

for(j in 1:3){

for(k in 2:2){

se[i,j,k] <- pow(se2[i,j,k],0.5)

se2[i,j,k] ~ dunif(0, 0.2)

}

}

}

for(i in c(2,4,5)){

for(j in 1:3){

for(k in 3:3){

se[i,j,k] <- pow(se2[i,j,k],0.5)

se2[i,j,k] ~ dunif(0, 0.2)

}

}

}

for(i in c(2:4)){

for(j in 1:3){

for(k in 4:4){

se[i,j,k] <- pow(se2[i,j,k],0.5)

se2[i,j,k] ~ dunif(0, 0.2)

}

}

}

for(i in c(1,5)){

for(j in 1:3){

for(k in 5:5){

se[i,j,k] <- pow(se2[i,j,k],0.5)

se2[i,j,k] ~ dunif(0, 0.2)

}

}

}

for(i in c(1,2,5)){

for(j in 1:3){

for(k in 6:6){

se[i,j,k] <- pow(se2[i,j,k],0.5)

se2[i,j,k] ~ dunif(0, 0.2)

}

}

}

for(i in 1:1){

for(j in 1:1){

for(k in 3:4){

se[i,j,k] <- pow(se2[i,j,k],0.5)

se2[i,j,k] ~ dunif(0, 0.2)

}

}

}

for(i in 2:2){

for(j in 1:1){

for(k in 5:5){

se[i,j,k] <- pow(se2[i,j,k],0.5)

se2[i,j,k] ~ dunif(0, 0.2)

}

}

}

# Random study effect distribution

for(i in 1:N){

b0[i] ~ dnorm(mu.b0,inv.tau2)

}

#Random outcome effects distributions

for(k in 1:P){

b1[k] ~ dnorm(mu.b1,inv.delta2)

b2[k] ~ dnorm(mu.b2,inv.gamma2)}

# Precisions

inv.sigma2 <- 1/sigma2

inv.tau2 <- 1/tau2

inv.delta2 <- 1/delta2

inv.gamma2 <- 1/gamma2

# Priors

sigma2 ~ dunif(0, 0.005) # residual variance

tau2 ~ dunif(0, 0.005) # random intercept variance (study)

delta2 ~ dunif(0, 0.005) # random slope variance

gamma2 ~ dunif(0, 0.005) # random intercept variance (outcome)

mu.b0 ~ dnorm(0,0.001) # mean beta0 coefficient

mu.b1 ~ dnorm(0,0.001) # mean beta1 coefficient

mu.b2 ~ dnorm(0,0.001) # mean beta2 coefficient

#PREDICTIONS #############

# Slope of eczema exposure

slope <- mu.b1

slope.pi ~ dnorm(mu.b1, inv.delta2)

}"

jdat2 <- list(N=length(levels(d3$study)), M=length(levels(d3$exposure)), P=length(levels(d3$outcome)),

logor=array(d3$logor, dim = c(length(levels(d3$study)),

length(levels(d3$exposure)),

length(levels(d3$outcome)))),

se=array(d3$se, dim = c(length(levels(d3$study)),

length(levels(d3$exposure)),

length(levels(d3$outcome)))),

exposure=1:length(levels(d3$exposure)))

set.seed(1000399)

jmod2 <- jags.model(textConnection(jcode2), data=jdat2, n.chains=2, n.adapt=1000)

update(jmod2,5000)

jpos2 <- coda.samples(jmod2, c("slope","slope.pi"), n.iter=100000, thin=5)

**References:**

1. Andersen YM, Egeberg A, Gislason GH, Hansen PR, Skov L, Thyssen JP. Risk of myocardial infarction, ischemic stroke, and cardiovascular death in patients with atopic dermatitis. J Allergy Clin Immunol 2016; 138:310-2 e3.

2. Drucker AM, Li WQ, Cho E, Li T, Sun Q, Camargo CA, Jr., et al. Atopic dermatitis is not independently associated with nonfatal myocardial infarction or stroke among US women. Allergy 2016; 71:1496-500.

3. Drucker AM, Qureshi AA, Dummer TJB, Parker L, Li WQ. Atopic dermatitis and risk of hypertension, type 2 diabetes, myocardial infarction and stroke in a cross-sectional analysis from the Canadian Partnership for Tomorrow Project. Br J Dermatol 2017; 177:1043-51.

4. Egeberg A, Andersen YM, Gislason GH, Skov L, Thyssen JP. Prevalence of comorbidity and associated risk factors in adults with atopic dermatitis. Allergy 2017; 72:783-91.

5. Kwa MC, Silverberg JI. Association Between Inflammatory Skin Disease and Cardiovascular and Cerebrovascular Co-Morbidities in US Adults: Analysis of Nationwide Inpatient Sample Data. Am J Clin Dermatol 2017; 18:813-23.

6. Marshall VD, Moustafa F, Hawkins SD, Balkrishnan R, Feldman SR. Cardiovascular Disease Outcomes Associated with Three Major Inflammatory Dermatologic Diseases: A Propensity-Matched Case Control Study. Dermatol Ther (Heidelb) 2016; 6:649-58.

7. Radtke MA, Schafer I, Glaeske G, Jacobi A, Augustin M. Prevalence and comorbidities in adults with psoriasis compared to atopic eczema. J Eur Acad Dermatol Venereol 2017; 31:151-7.

8. Riis JL, Vestergaard C, Hjuler KF, Iversen L, Jakobsen L, Deleuran MS, et al. Hospital-diagnosed atopic dermatitis and long-term risk of myocardial infarction: a population-based follow-up study. BMJ Open 2016; 6:e011870.

9. Silverberg JI. Association between adult atopic dermatitis, cardiovascular disease, and increased heart attacks in three population-based studies. Allergy 2015; 70:1300-8.

10. Silverwood RJ, Forbes HJ, Abuabara K, Ascott A, Schmidt M, Schmidt SAJ, et al. Severe and predominantly active atopic eczema in adulthood and long term risk of cardiovascular disease: population based cohort study. BMJ 2018; 361:k1786.

11. Standl M, Tesch F, Baurecht H, Rodriguez E, Muller-Nurasyid M, Gieger C, et al. Association of Atopic Dermatitis with Cardiovascular Risk Factors and Diseases. J Invest Dermatol 2017; 137:1074-81.

12. Su VY, Chen TJ, Yeh CM, Chou KT, Hung MH, Chu SY, et al. Atopic dermatitis and risk of ischemic stroke: a nationwide population-based study. Ann Med 2014; 46:84-9.

13. Sung YF, C. LC, H. YJ, Chou CH, Chung CH, Yang FC, et al. Increased Risk of Stroke in Patients with Atopic Dermatitis: A Population‑based, Longitudinal Study in Taiwan. Journal of Medical Sciences 2017; 2017:12-8.

14. Treudler R, Zeynalova S, Walther F, Engel C, Simon JC. Atopic dermatitis is associated with autoimmune but not with cardiovascular comorbidities in a random sample of the general population in Leipzig, Germany. J Eur Acad Dermatol Venereol 2018; 32:e44-e6.

15. Tsai KS, Yen CS, Wu PY, Chiang JH, Shen JL, Yang CH, et al. Traditional Chinese Medicine Decreases the Stroke Risk of Systemic Corticosteroid Treatment in Dermatitis: A Nationwide Population-Based Study. Evid Based Complement Alternat Med 2015; 2015:543517.

16. Varbo A, Nordestgaard BG, Benn M. Filaggrin loss-of-function mutations as risk factors for ischemic stroke in the general population. J Thromb Haemost 2017; 15:624-35.

17. DerSimonian R, Laird N. Meta-analysis in clinical trials. Control Clin Trials 1986; 7:177-88.

18. Higgins JP, Thompson SG, Deeks JJ, Altman DG. Measuring inconsistency in meta-analyses. BMJ 2003; 327:557-60.

19. Guddat C, Grouven U, Bender R, Skipka G. A note on the graphical presentation of prediction intervals in random-effects meta-analyses. Syst Rev 2012; 1:34.

20. van de Schoot R, Depaoli S. Bayesian analyses: where to start and what to report. Health Psychol Rev 2014; 16:75-84.

21. R Core Team. R: A language and environment for statistical computing. Vienna, Austria: R Foundation for Statistical Computing; 2017.] Available from [https://www.r-project.org/](https://www.R-project.org/).

22. Schwarzer G. meta: An R package for meta-analysis. R News 2007; 7:40-5.

23. Plummer M. rjags: Bayesian Graphical Models using MCMC. R package version 4-6., 2016.
